# Supplementary material for: Early progression to active tuberculosis is a highly heritable trait driven by 3q23 in Peruvians
Source: Nat Commun. 2019 Aug 21;10:3765. doi: 10.1038/s41467-019-11664-1 (PMC6704092; doi:10.1038/s41467-019-11664-1)
Supplement: Supplementary file 1 — Supplementary Information [file 41467_2019_11664_MOESM1_ESM.pdf]

Luo et al. “Early progression to active tuberculosis is a highly heritable trait driven by 3q23 in Peruvians”

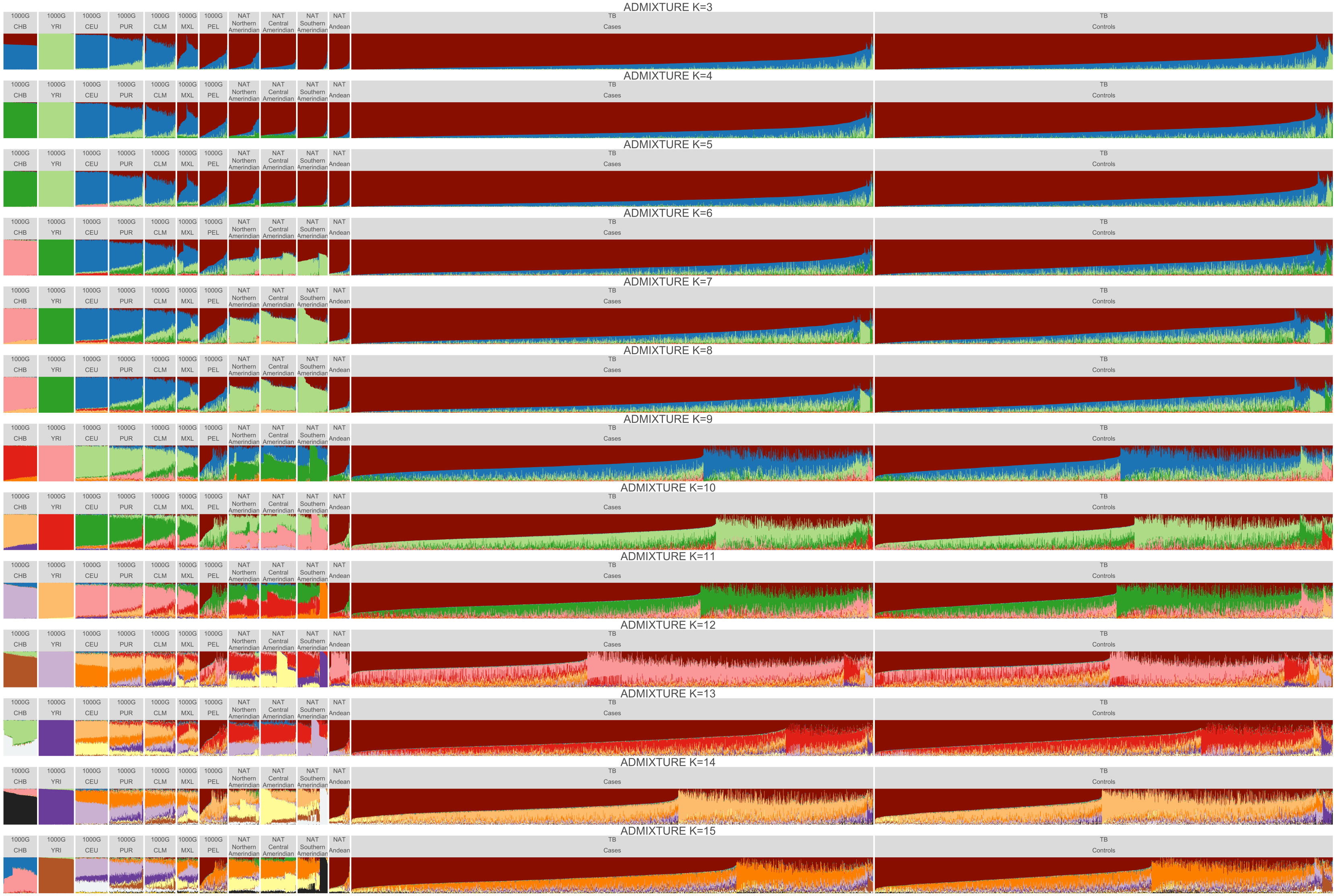

Supplementary Figure 1. ADMIXTURE plot of Peruvian individuals and other populations. Each individual is represented as a thin vertical bar. The colors represented the proportion of ancestry assigned to each cluster for each individual. K=3 through K=15 models are shown. Reference panels are either from the 1000 Genomes project (1000G) or Native American individuals collected from *Reich et al. 2012 Nature* (NAT). CHB represents Han Chinese in Beijing, China; YRI represents Yoruba in Ibadan, Nigeria; CEU represents Utah Residents (CEPH) with Northern and Western European Ancestry; PUR represents Puerto Ricans from Puerto Rico; CLM represents Colombians from Medellin, Colombia; MXL represents Mexicans from Los Angeles, California; PEL represents Peruvians from Lima, Peru. Northern Amerindian includes individuals from Maya, Mixe and Kaqchikel. Central Amerindian includes individuals from Pima, Zapotec, Mixtec, Yaqui, Chorotega, Tepehuano. Southern Amerindian includes individuals from Piapoco, Karitiana, Surui, Wayuu, Jamamadi, Parakana, Guarani, Kaingang, Ticuna, Palikur, Toba, Arara, Wichi, Chane and Guahibo. Andean population includes Quechua and Aymara. Source data are provided as a Source Data file.

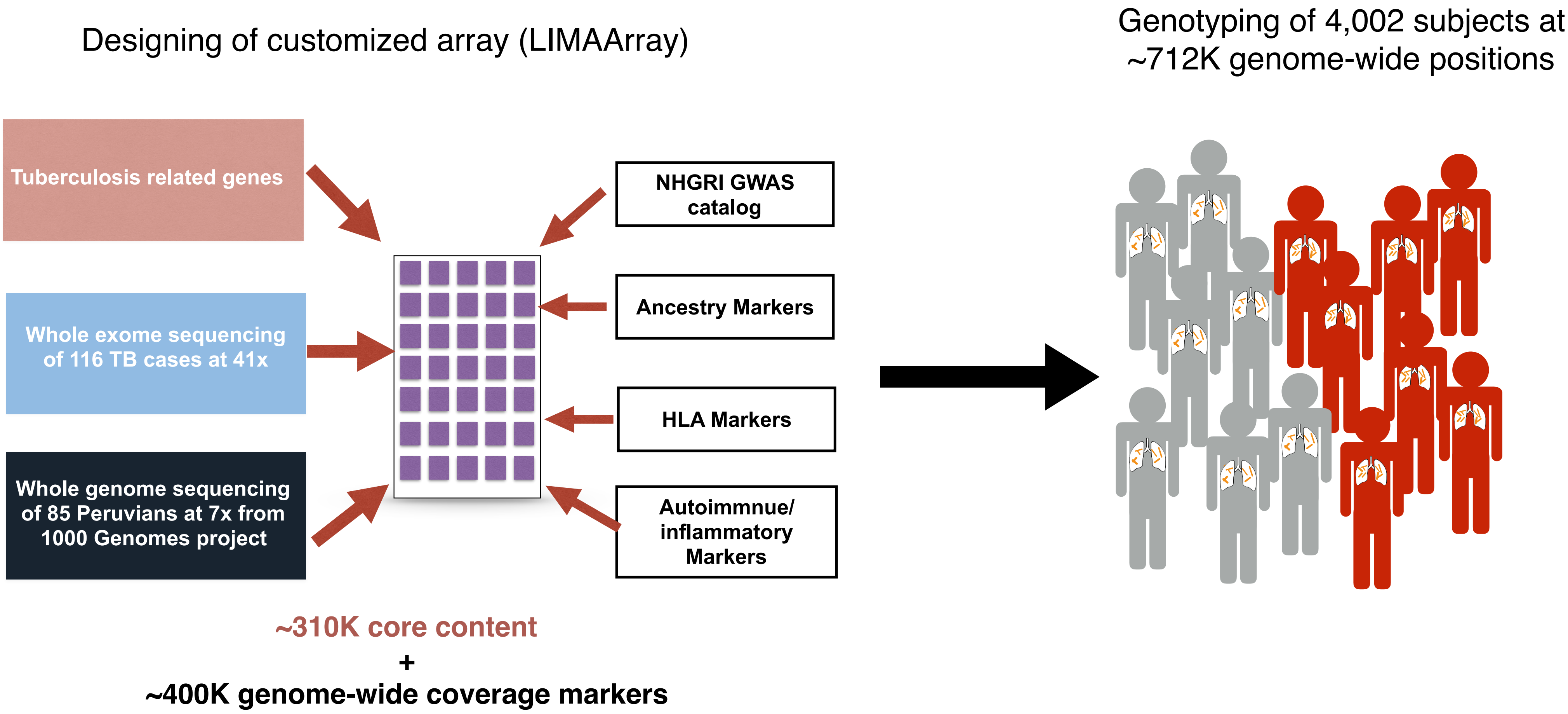

Supplementary Figure 2. LIMAArray design pipeline. A cartoon illustrates genome-wide array design tailored for the Peruvian population.

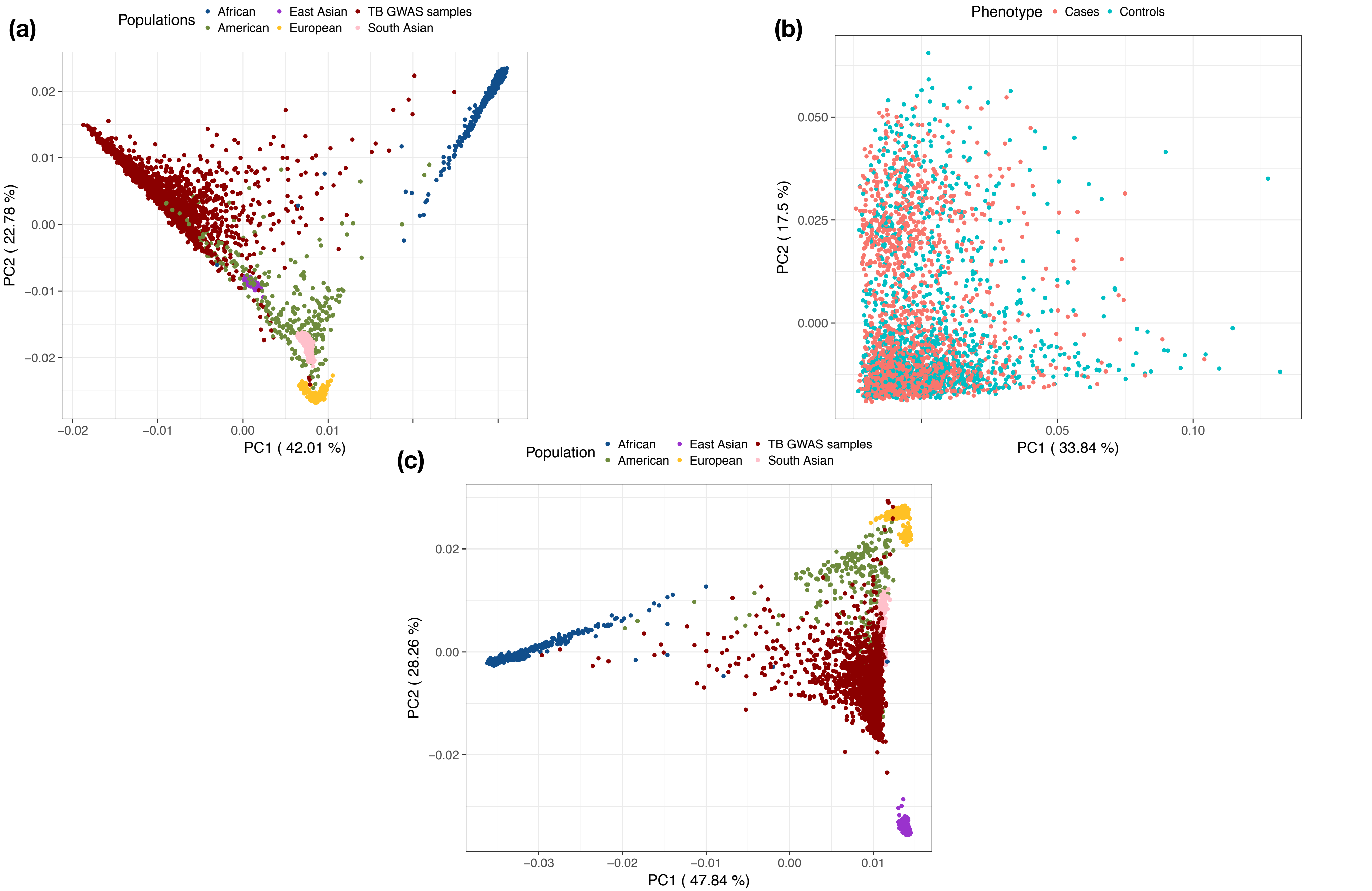

Supplementary Figure 3. Principal component analysis of the GWAS samples. (a) First and second principal components. Peruvian samples are plotted with five 1000 Genomes Phase 3 populations. (b) All GWAS samples plotted on the first two principal components colored by the disease status. (c) TB cases and controls projected onto the first two principal components using SNP weights precomputed from samples in the 1000 Genomes Phase 3 project using SNPweights. Source data are provided as a Source Data file.

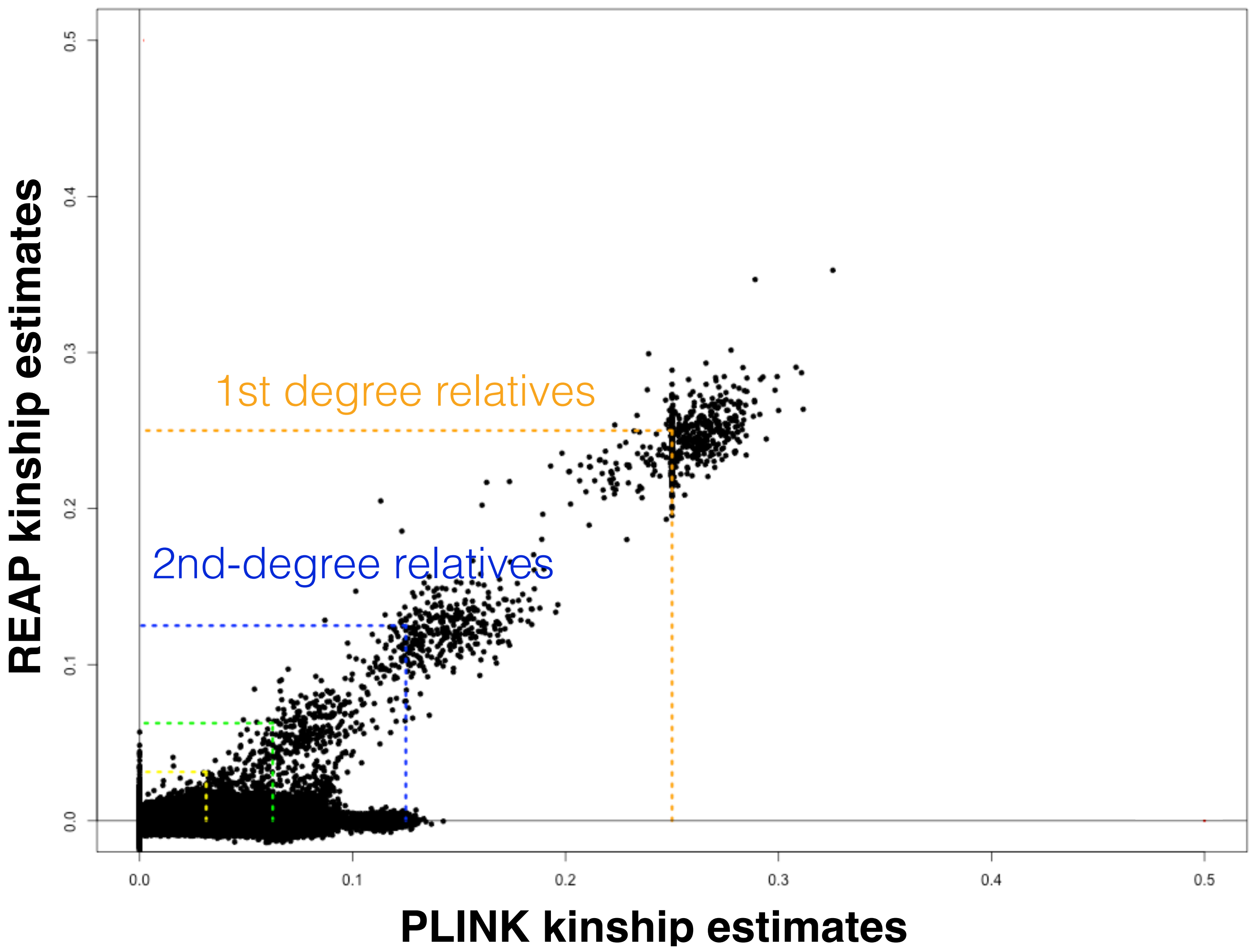

Supplementary Figure 4. Kinship estimates using REAP and PLINK. Relative pairs were classified on the basis of kinship-coefficient estimates based on two different softwares. REAP (Relatedness Estimation in Admixed Populations) is a program that accounts for ancestry among sample individuals to estimate autosomal kinship coefficients using genome-wide SNP genotype data due only to recent family structure. Source data are provided as a Source Data file.

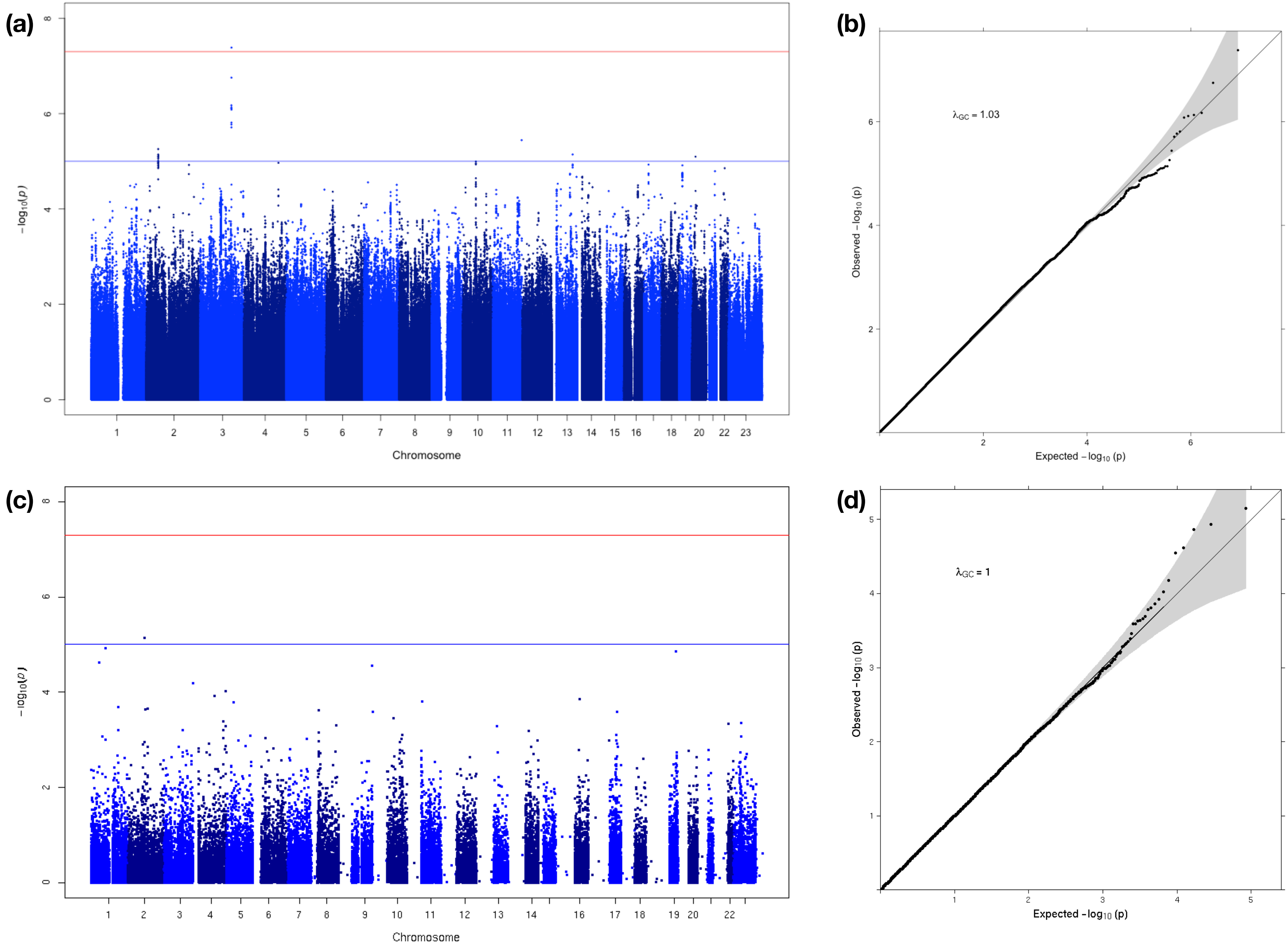

Supplementary Figure 5. Manhattan and QQ-plots of TB progression for 7,756,401 variants after genotype imputation. Manhattan plot showing genome-wide association study for (a) single common variants (6,035,269, MAF>=1%) and (c) rare variant (1,721,132, MAF<1%) burden analysis. QQ-plot of common variant association study (b) and rare variant burden analysis (d). The diagonal black line in all QQ-plots is y = x, and the grey shapes show 95% confidence interval under the null. s are the genome-wide inflation factors based on all tested statistics. Source data of the common variants association results are available from GWAS Catalog (see Data Availability). Source data of the rare variant burden results are provided as a Source Data file.

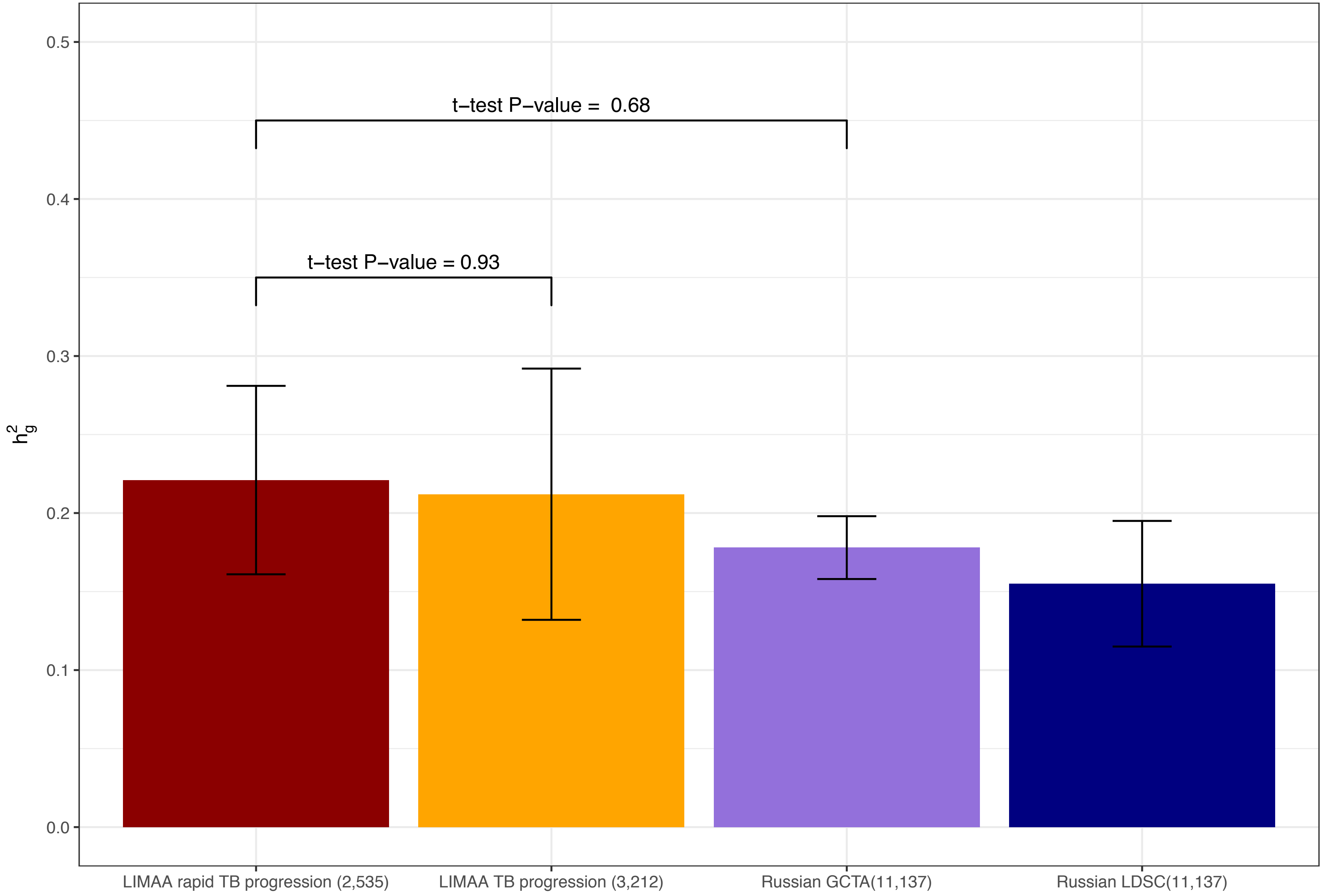

Supplementary Figure 6. Heritability estimates of TB progression and population-wide TB susceptibility. Each bar plot represents the genetic heritability estimates (h_g^2) and its standard error based on different cohort definition and statistical method that had been employed as described in the x-axis. P-values are derived from the student t distribution. The number of samples used in each estimation is reported in the bracket. Source data are provided as a Source Data file.

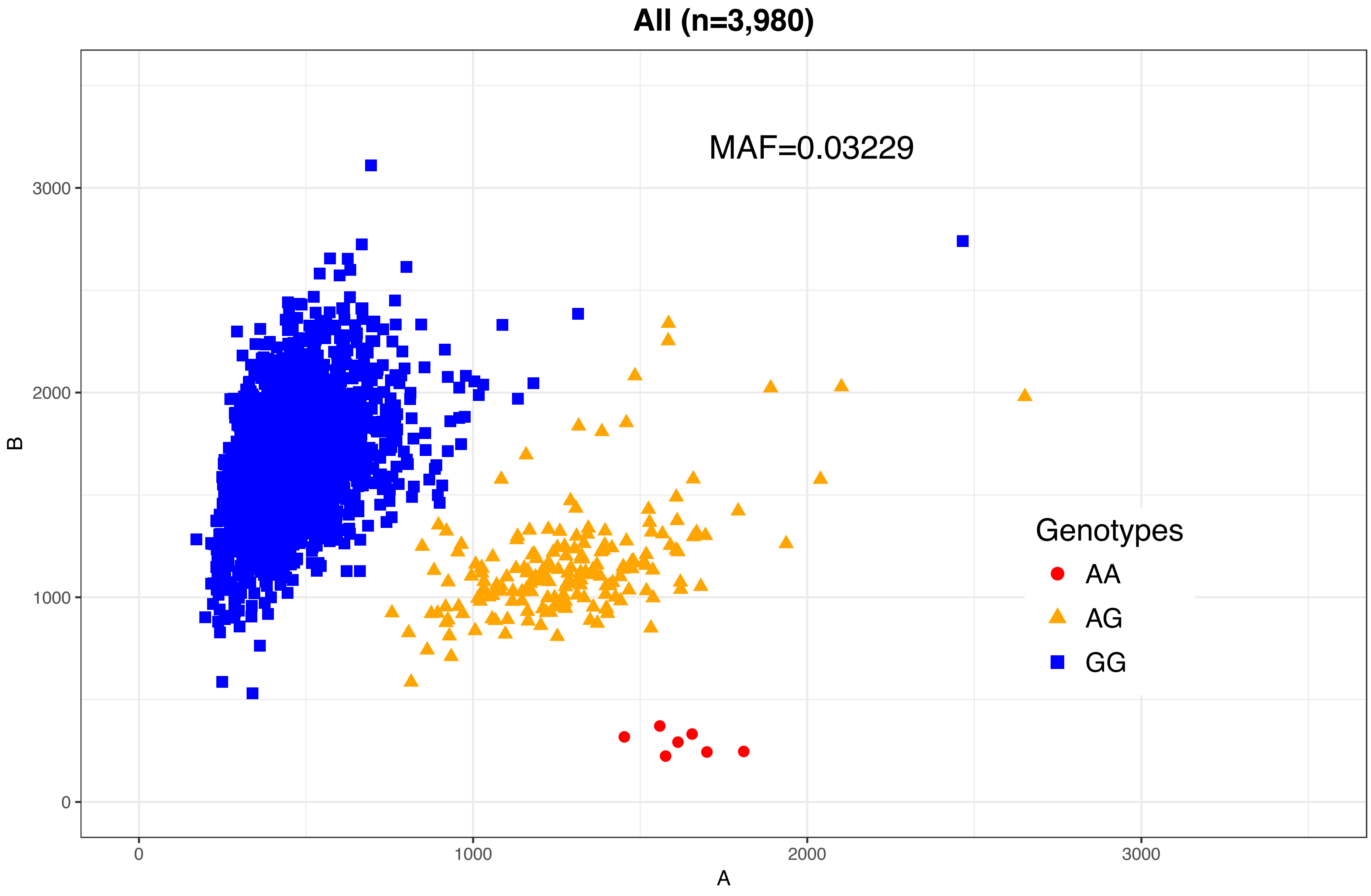

Supplementary Figure 7. Intensity cluster plot of SNP rs73226617. The SNP genotypes have been assigned based on cluster formation in scatter plots of normalized allele intensities A and B. Source data are provided as a Source Data file.

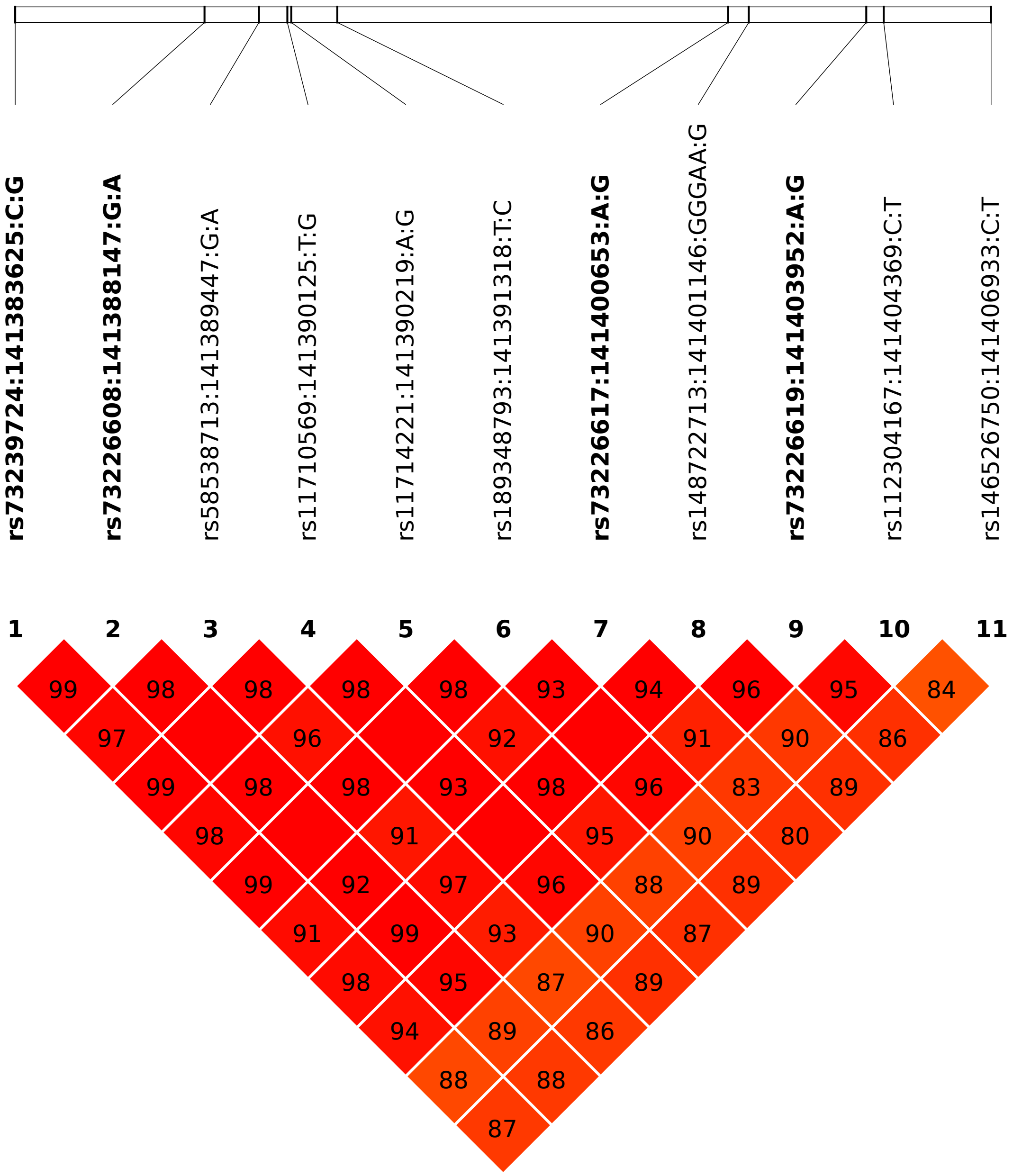

Supplementary Figure 8. Haploview LD graph of the reported top associations. Pairwise LD coefficients r^2 are shown in each cell (r^2 values of 1.0 are not shown). Source data are provided as a Source Data file.

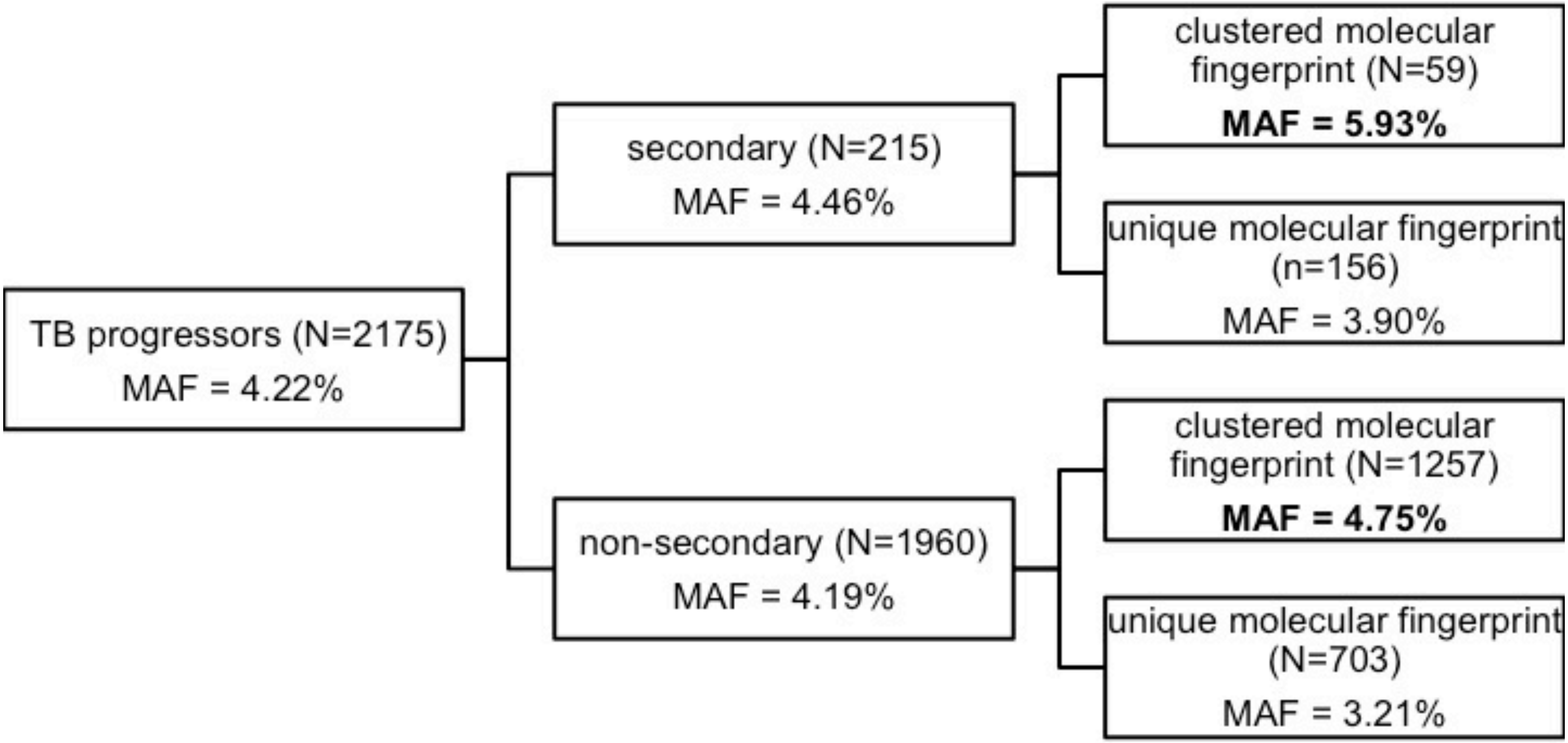

Supplementary Figure 9. TB cases stratified by a molecular fingerprint. All cultures of the cases were genotyped using MIRU-VNTR. TB cases share the same molecular fingerprint are epidemiologically more related while cases in which fingerprints are unique are due to remote infection that has reactivated. Reported minor allele frequency (MAF) in each category is of the top associated variant rs73226617.

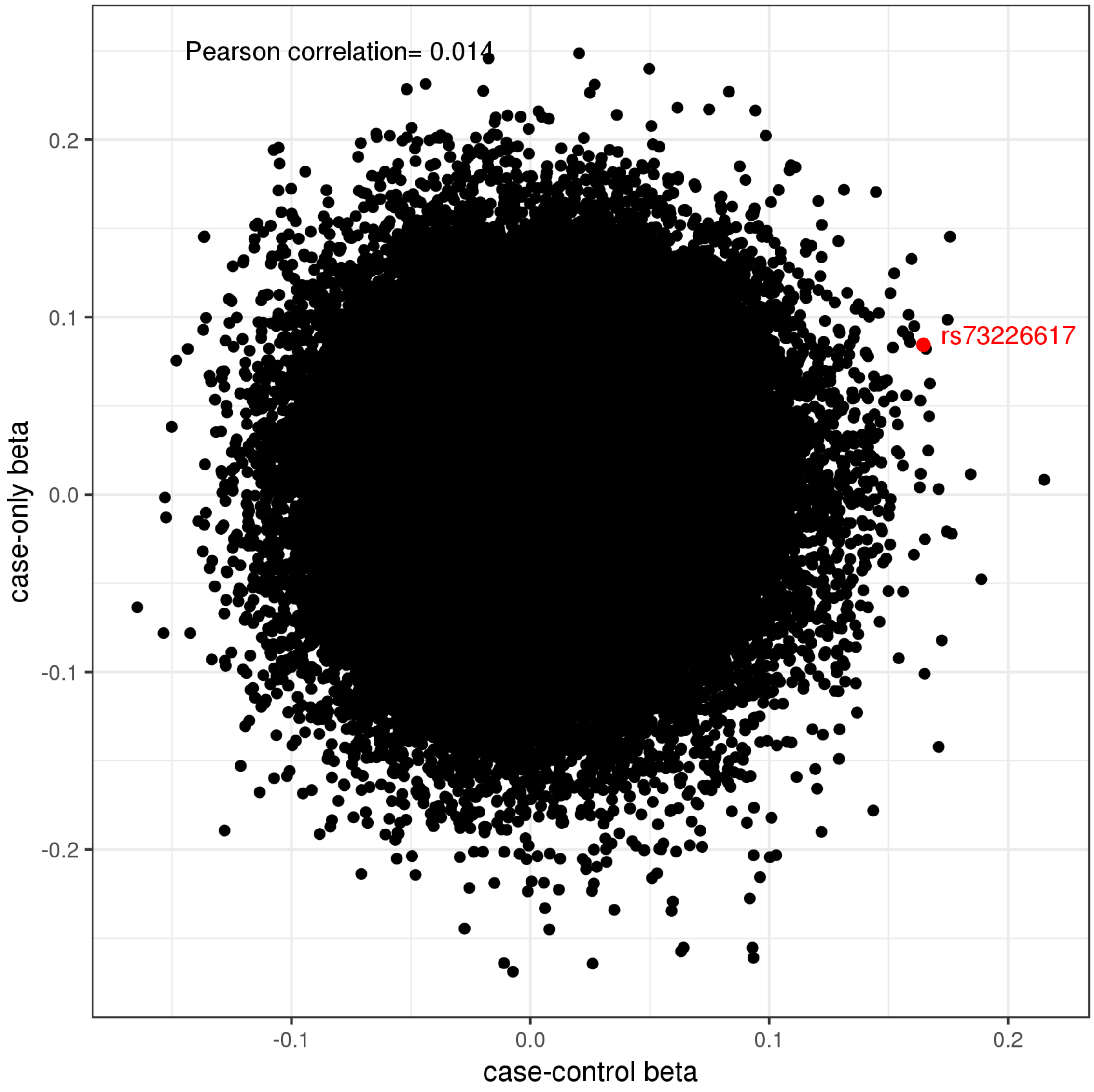

Supplementary Figure 10. Correlation between effect size (beta) between case-control (active TB cases versus latent TB controls) analysis and within case (early progressors versus other TB cases). Each dot in the plot represents a genetic variant, if the two tests are dependent, then there should be a non-zero correlation between two betas. Instead, we observed a Pearson correlation (r) of 0.014, suggesting the secondary, within case-only, analysis can be considered as independent test compared to the primary (case-control) analysis. The SNP (rs73226617) highlighted in red is the top associated risk variant. Source data are provided as a Source Data file.

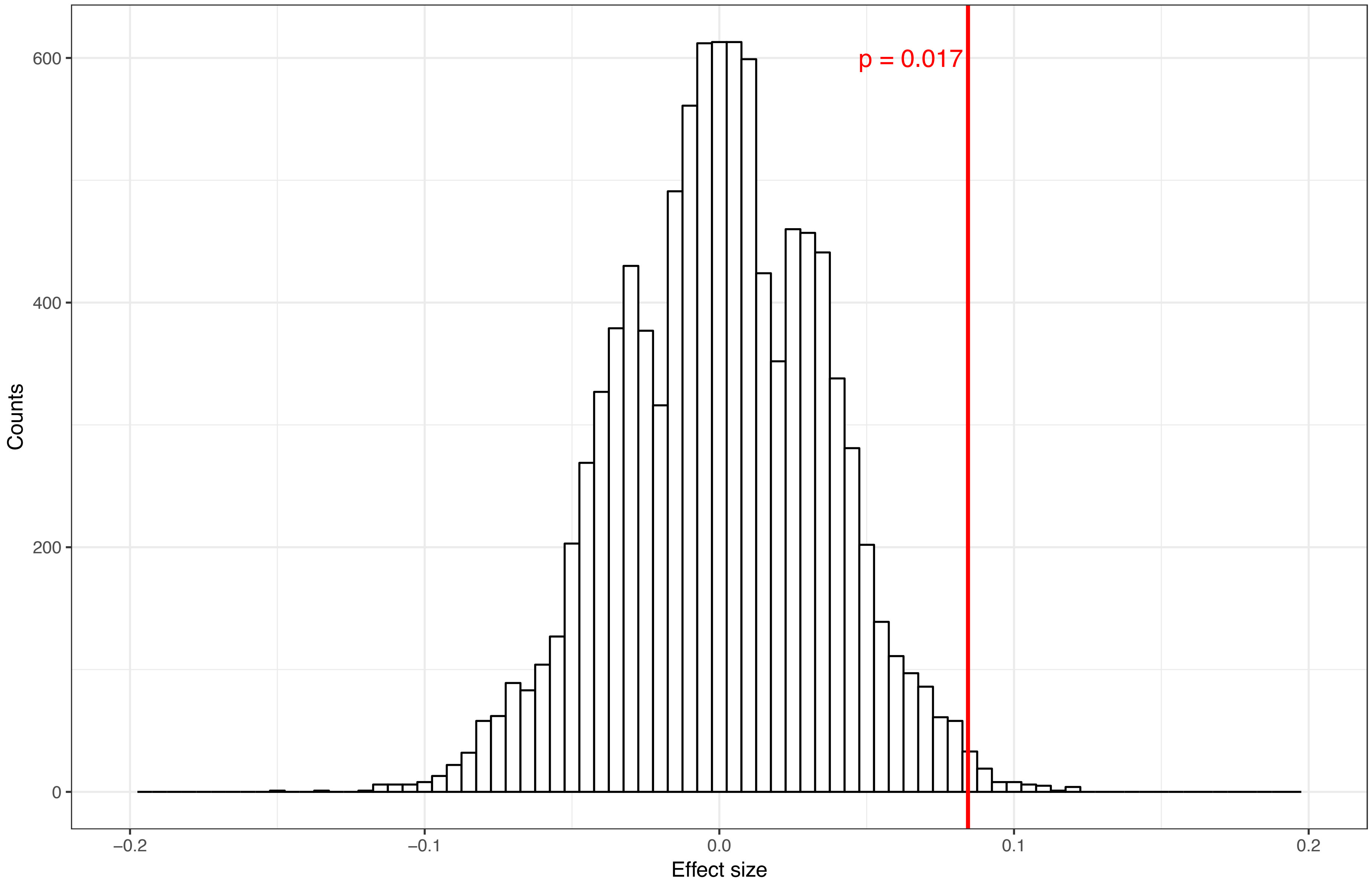

Supplementary Figure 11. Random permutation test of individuals in early and other progressors among active TB cases. The distribution of effect size was generated by randomly assigning early and other status among 2,160 TB cases. The red line in the panel marks the actual effect size observed. We conclude the observed OR of 1.09 has a P-value of 0.017 compared to null. That is only 1.7% of the observations have an effect size greater than the observed value (0.084, red vertical line). Source data are provided as a Source Data file.

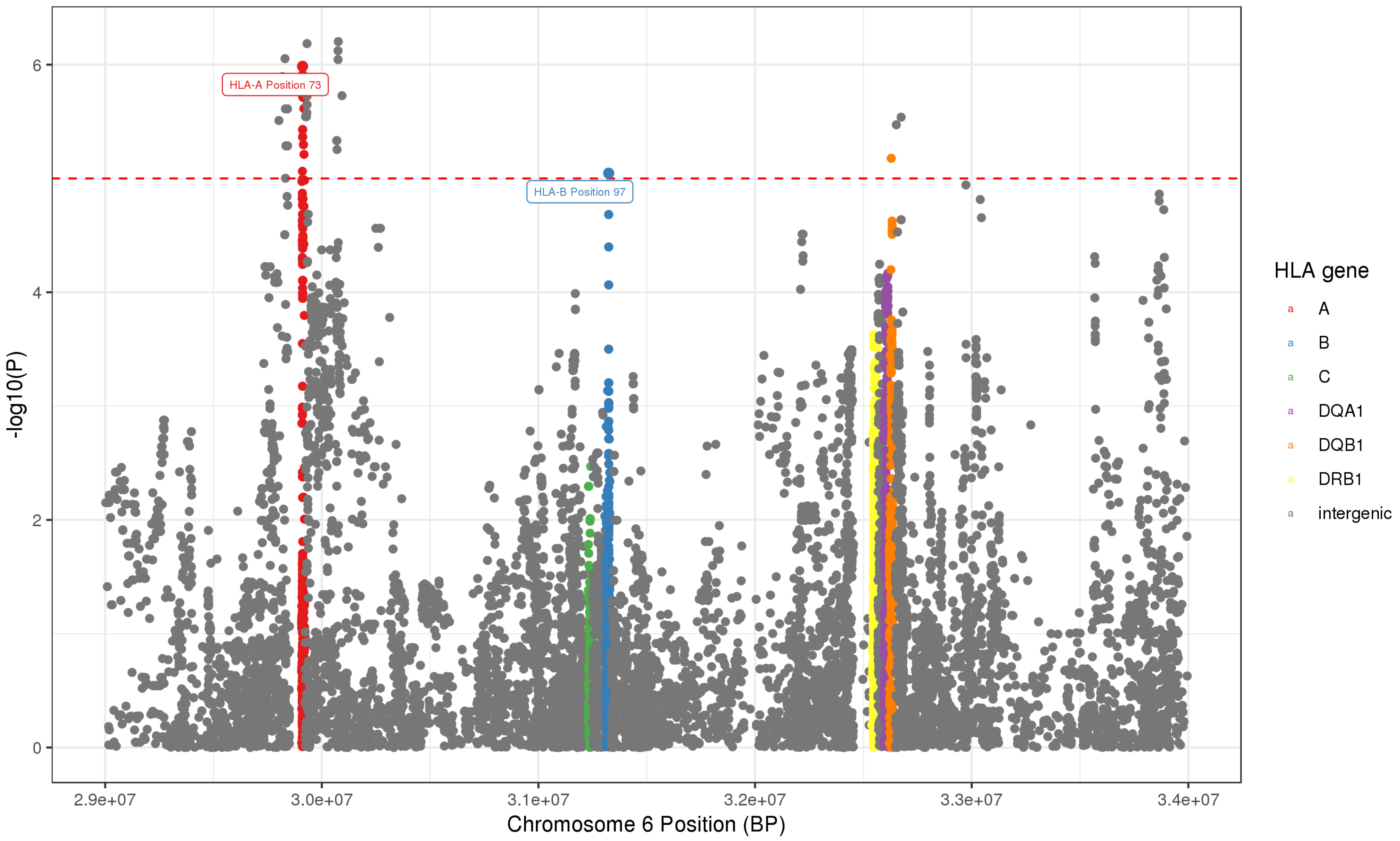

Supplementary Figure 12. Manhattan plot of HLA region. We imputed HLA region using SNP2HLA with a multi-ethnic HLA reference panel. The most significant amino acid association is position 73 of HLA-A (OR=1.12, $P = 1.03 \times 10^{-6}$). Source data are provided as a Source Data file.

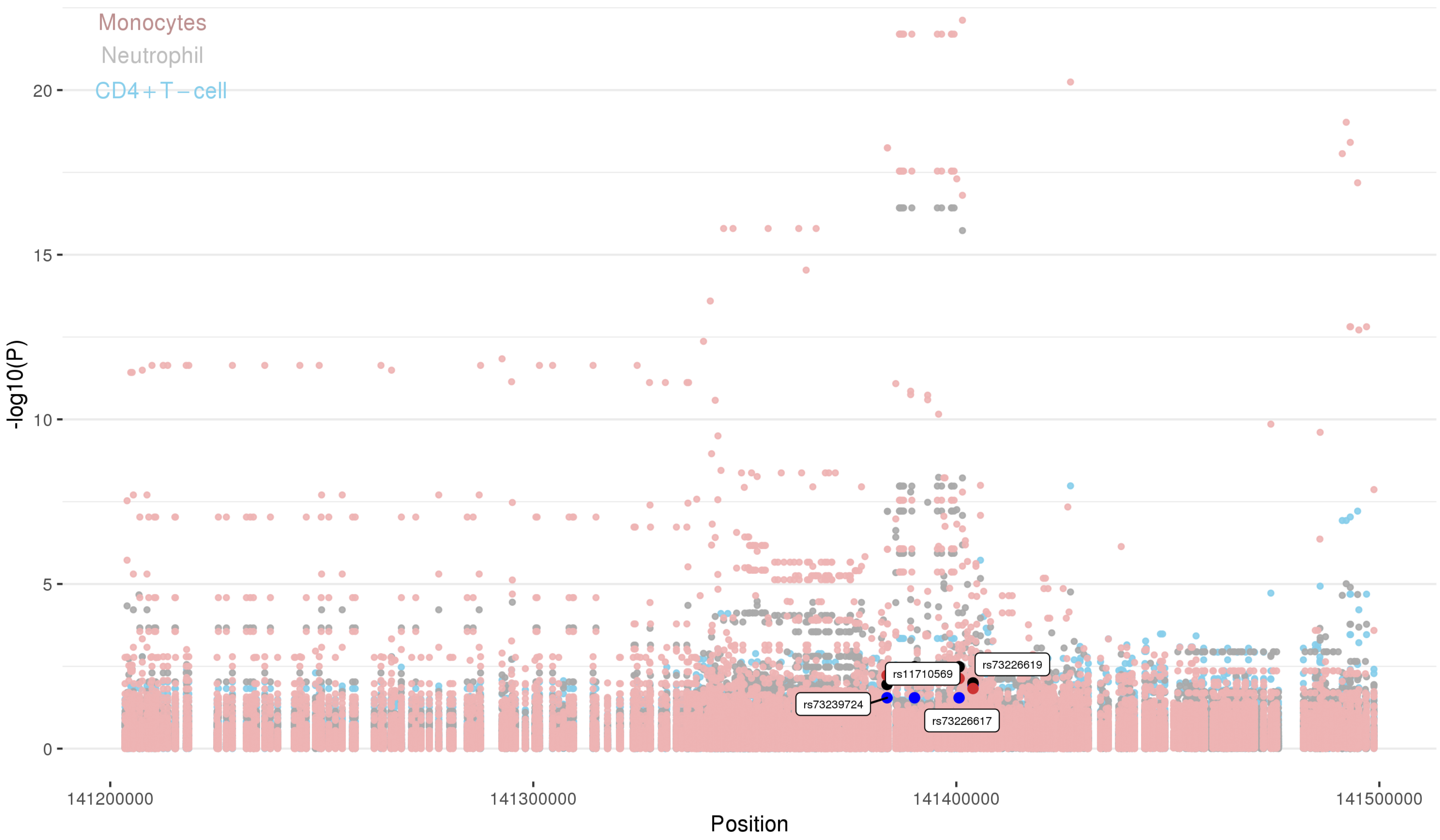

Supplementary Figure 13. Chromatin QTL analysis results in the Blueprint project. To understand the effects of genetic variants in immune cells, we utilized eQTL summary statistics produced by Blueprint project. Detailed methods were reported in the original article. Briefly, CD14+ monocytes (brown), CD16+ neutrophils (grey), and naive CD4+ T cells (light blue) were collected from 197 individuals, histone variation (H3K4me1) were analyzed. Genetic variants within 1 Mb of each feature were tested their association with normalized features using linear regression model including a random effect term accounting for sample relatedness. Four top risk variants that are associated with TB progression were included in the analysis (annotated in white boxes). Source data are provided as a Source Data file.

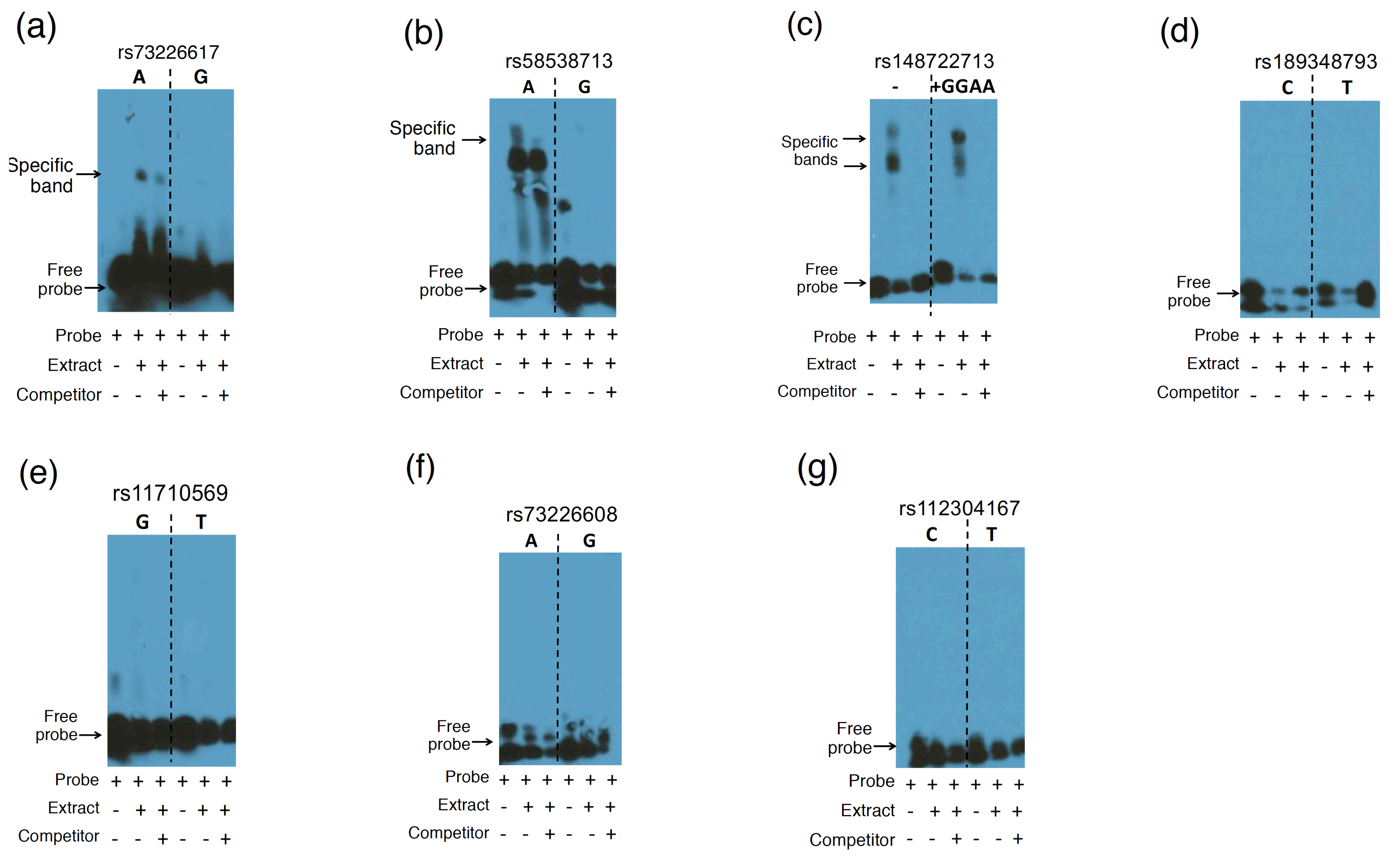

Supplementary Figure 14. EMSA for top seven associated variants. (a) rs73226617 (b) rs58538713 (c) rs148722713 (d) rs189348793 (e) rs11710569 (f) rs73226608 (g) rs146526750. Lanes in the panel correspond to double stranded probes without (lane 1) or with THP1 nuclear extracts (lane2) and an additional non-biotinylated competitor probe (lane 3). The experiment was performed on three independent batches of THP1 nuclear extracts. Uncropped versions of all gels are provided as a Source Data file.

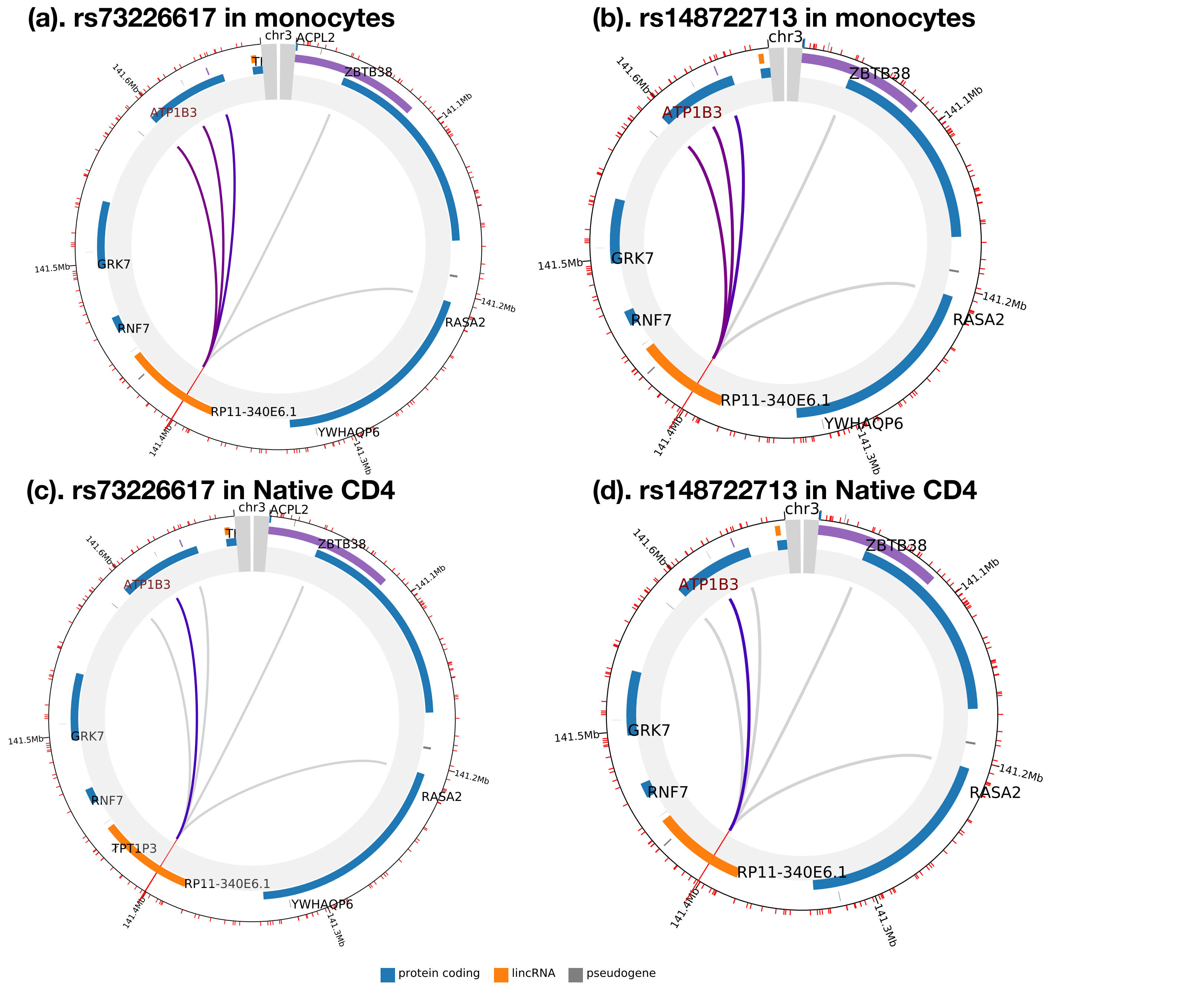

Supplementary Figure 15. Promoter capture Hi-C from www.chicp.org. Selected public promoter Hi-C data in 17 human primary hematopoietic cell types reveals (a)-(b) strong monocyte interactions (highest score = 9.54) between an enhancer region containing the leading risk variant (rs73226617) and *ATP1B3* in monocyte. This interaction is much weaker in (c)-(d) the Naive CD4+ T cells and other cell types (highest score = 5.51).

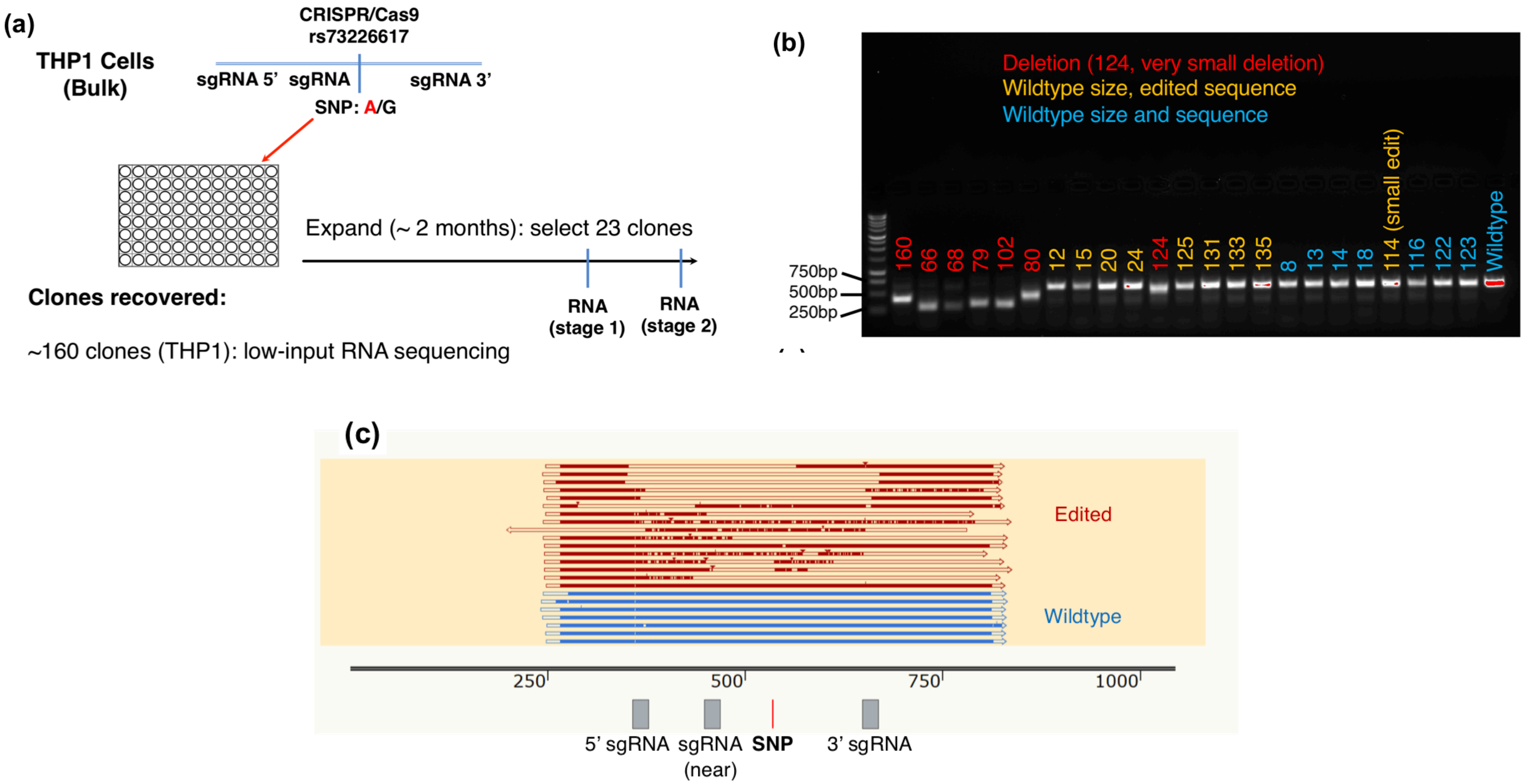

Supplementary Figure 16. Overview of the CRISPR/Cas9 experiment. (a) CRISPR/Cas9 strategy to disrupt the enhancer region surrounding the rs73226617 lead risk variant in 3q23. THP1 cells were nucleofected with 3 guide RNA molecules targeting genomic region around the variant, then expanded for RNA extractions and gene expression analysis. Bulk-edited THP1 cells were also single-cell sorted into 96 well-plates and expanded for DNA extractions and sanger sequencing for initial screening. 23 clones were expanded to represent different edits, where some show evidence of genomic deletion, or intact sequence length, for gene expression analysis by low-input RNA sequencing and qRT-PCR. (b) Amplicons were analyzed by gel electrophoresis to confirm deletions detected after initial screening. Intact amplicons are expected around 700 base pairs (wildtype band, far left). (c) Alignment of sanger sequences derived from the 23 THP1 clones showing location of edits compared to wildtype (unedited) amplicon sequences. Red and blue sequences represent edited and unedited THP1 clones, respectively.

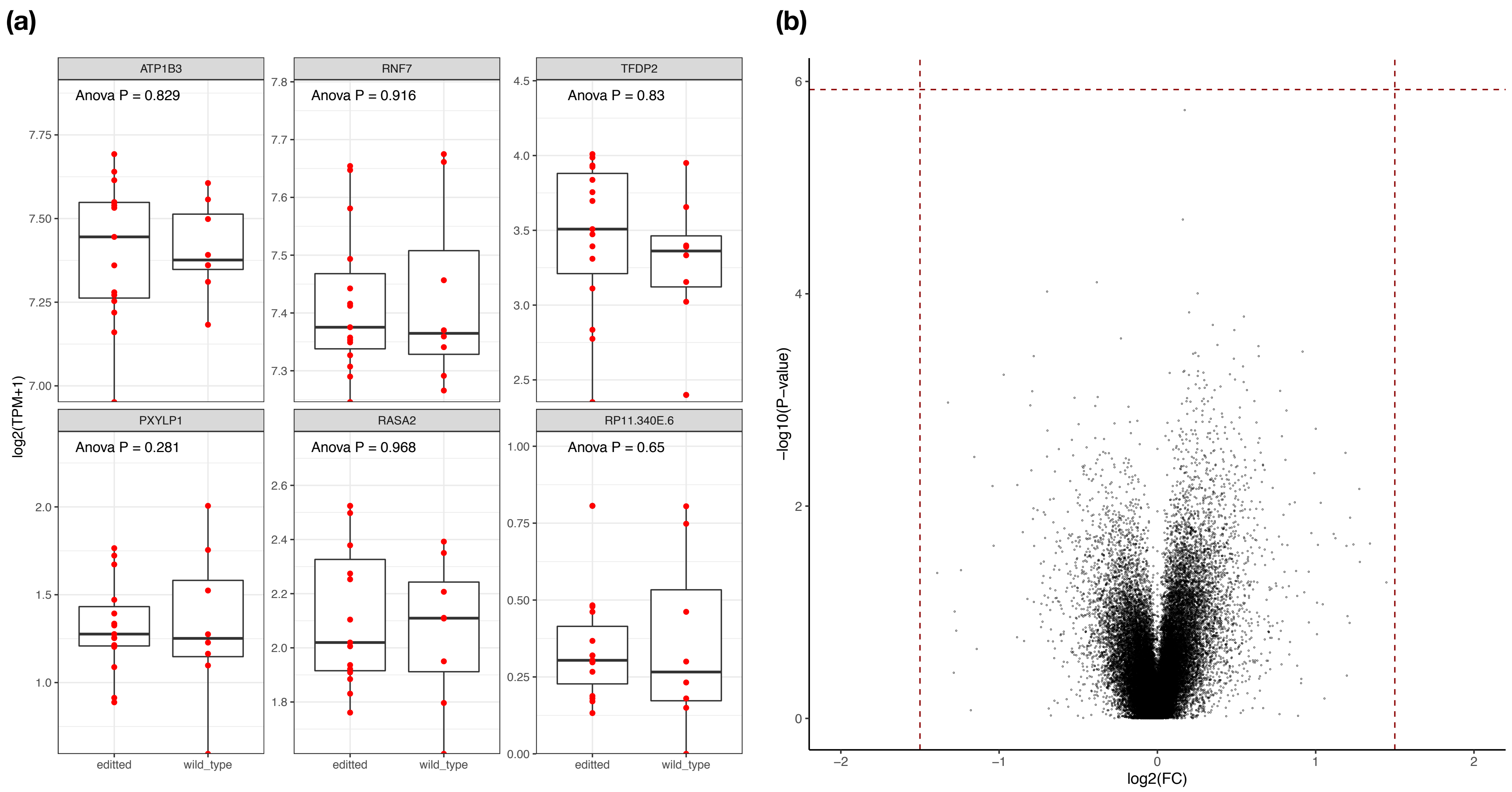

Supplementary Figure 17. Low-input RNA-sequencing analysis. (a) Expression of six genes around rs73226617 with transcripts per million (TPM) >1 in THP1 clones, which maintained wildtype genomic sequence after expansion of single cells from bulk-edited THP1 cells compared to edited clones. P-values are derived from a linear regression model including first principal component of the gene expression profile as covariate. (b) Volcano plot from RNA-seq data showcasing global expression of transcripts enriched in wildtype (left, n=7) or edited (right, n=16) THP1 clones. Source data are provided as a Source Data file.

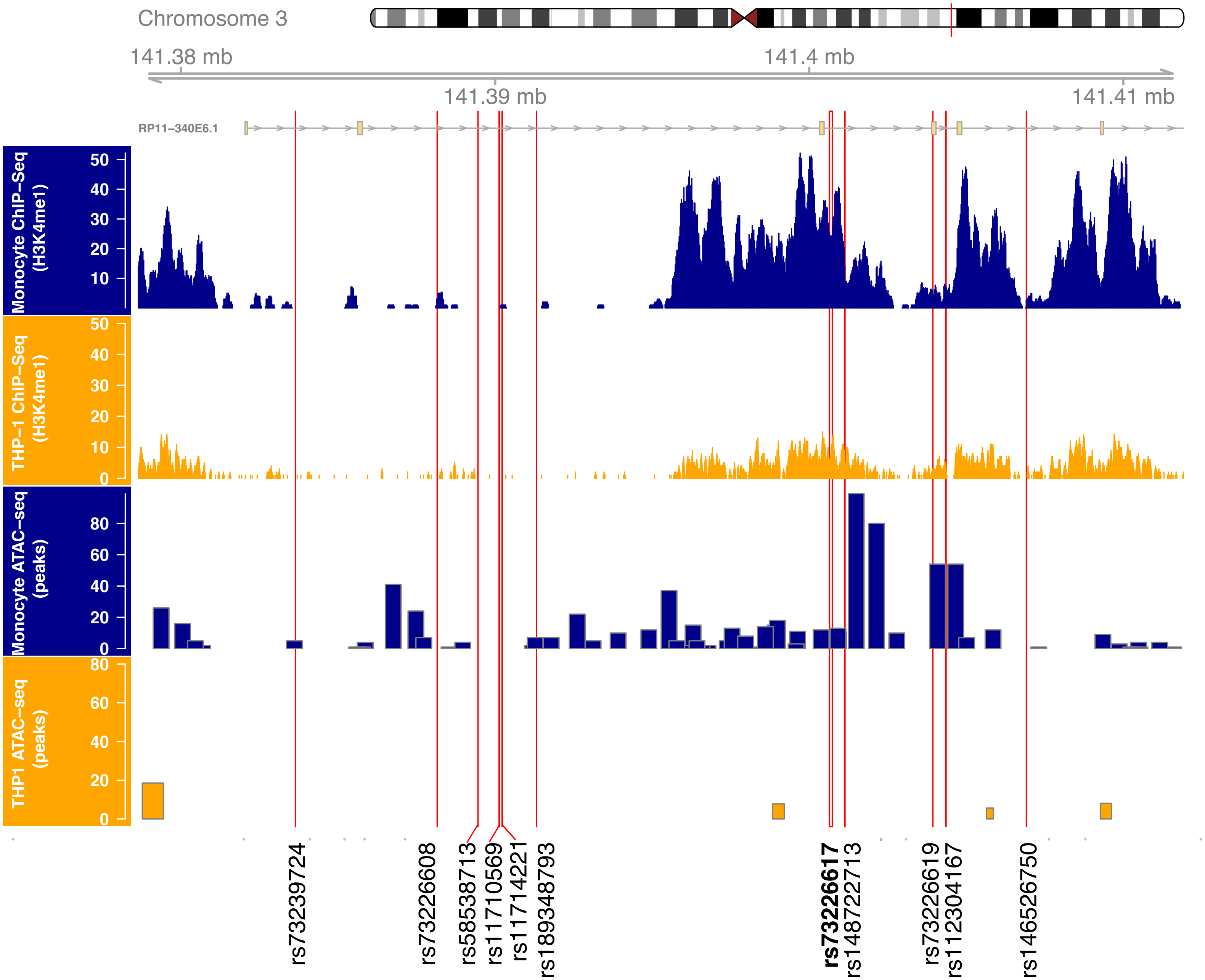

Supplementary Figure 18. Enhancer activity of the risk locus (3q23) in primary monocytes and THP1 cell lines indicated by ChIP-seq and ATAC-seq. From top to bottom, the y-axis shows the raw reads of ChIP-seq for H3K4me1 in primary monocytes (GSM1003535) and in THP-1 cell lines (GSM3514950); raw counts of ATAC-seq in primary monocytes (GSE74912) and in THP1 cell lines (GSE96800). The x-axis shows the genomic positions of the identified risk locus (chr3:141383525-141407033). The vertical lines highlights 11 top associated variants. Genotyped variant rs73226617 is highlighted in bold.

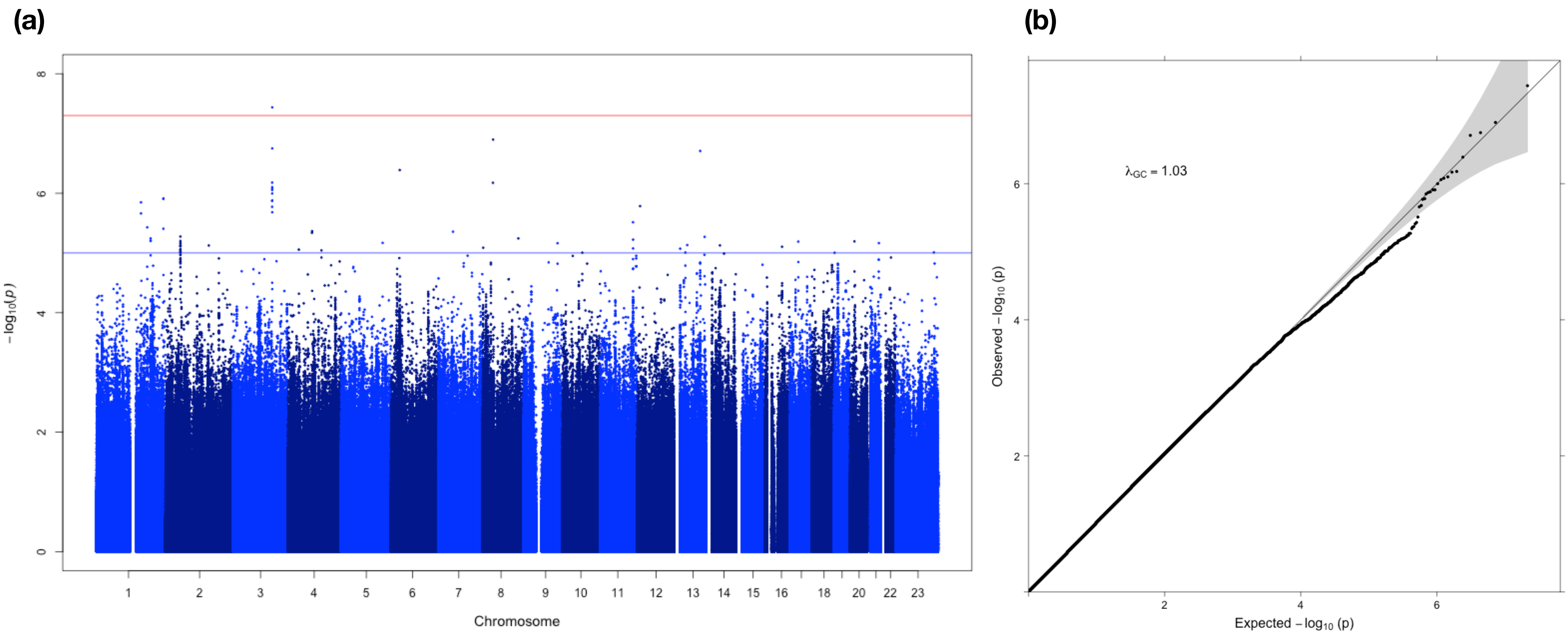

Supplementary Figure 19. Manhattan and QQ-plots of TB progression including the Native American proportions as a covariate in the linear mixed model. Manhattan (a) and QQ (b) plot showing genome-wide association study for single common variants (6,035,269, MAF>=1%). P-values were reported from the linear mixed model using the genetic relatedness matrix (GRM) as random effects. Sex, age and Native American proportions inferred from the ADMIXTURE analysis (K=6) were included as fixed effects. The diagonal black line in all QQ-plots is y = x, and the grey shapes show 95% confidence interval under the null. s are the genome-wide inflation factors based on all tested statistics. Source data are provided as a Source Data file.

Supplementary Table 1. GWAS cohort summary. Numbers are shown for all individuals with genotype data available. sd, standard deviation

	Total samples (N%)	Males (N%)	Females (N%)	Mean ages (sd)	Mean age in males (sd)	Mean age in females (sd)
**Cases**	2,175 (54.3%)	1,353 (59.3%)	822 (47.8%)	29.2 (13.1)	29.5 (13.4)	28.6 (12.4)
**Controls**	1,827 (45.7%)	929 (40.7%)	898 (52.2%)	33.1 (16.0)	32.6 (15.5)	33.5 (16.5)

Supplementary Table 2. LIMAA Affymetrix Axiom array design summary. Number of markers in each design module.

Module	Number_of_markers
GWAS/HLA/Immune-related	8.1K
Ancestry markers	4.5K
WES/WGS core markers	302.4K
Markers for genomic coverage	397.2K
Total	712.2K

Supplementary Table 3. Imputation quality of LIMAArray when evaluted against PEL (Peruvian) panel in 1000 Genomes project Phase 3.
Imputation quality indiciates by R2 using the IMPUTE2 software.

		LIMAArray			Axiom Biobank			UK Biobank		
**MAF range**	**n.target**	**MeanR2**	**frac.R2>=0.4**	**frac.R2>=0.8**	**MeanR2**	**frac.R2>=0.4**	**frac.R2>=0.8**	**MeanR2**	**frac.R2>=0.4**	**frac.R2>=0.8**
[0.005,0.01]	4989019	0.565	0.581	0.543	0.536	0.553	0.513	0.561	0.575	0.543
[0.01,0.05)	3467365	0.856	0.907	0.789	0.798	0.87	0.687	0.833	0.892	0.746
[0.05,0.5]	6569634	0.929	0.973	0.912	0.906	0.962	0.864	0.925	0.968	0.896

Supplementary Table 4. Heritability estimates using different methods when calculating genetic relatedness matrix.
In all scenarios, we cacluated GRMs after removing individuals (--grm-cutoff 0.125) and corrected for population stratifications (--qcovar) using top 10 principal components.

Method	heritability (hg2)	standard error	N
GCTA (0.125 unrelated)	0.212	0.080	3,179
GCTA (REAP GRM)	0.204	0.105	3,179
GCTA (PC-RELATE GRM)	0.181	0.072	3,179

Supplementary Table 5. Estimatates of h2g for GWAS traits. For comparison, we included six previously reported h2g using imputed GWAS data. All reported esitmates were under the liability scale with assume disease prevalence. TB susceptiblity are estimated using GCTA in a Russian GWAS dataset, and h2SNP of TB progression were obtained using GCTA after removing related individuals.

Trait	Prevalence	Imputed_hg2	standard_error	Source	#_gwas_loci	sample_size(1000)
Crohns disease	0.005	0.284	0.016	Luo et al. 2017	165	27
Rheumatoid arthritis	0.005	0.09	0.033	Gusev et al. 2015	101	57
Schizophrenia	0.01	0.18	0.024	Gusev et al. 2015	139	108
genome-wide TB susceptility	0.04	0.178	0.02	Curtis&Luo et al. 2015	1	11
early TB progression	0.1	0.212	0.08	This study	1	4
Leprosy	0.0001	0.199	0.01	Wang et al 2016	24	24
HIV-1 virus load		0.246	0.03	McLaren et al. 2015	2	6

Supplementary Table 7. Design details of Taqman genotyping assays.

SNP	Alleles	Context Sequence [VIC/FAM]
rs73239724	G/C	ACTTTCACAGTGCCTTTGCTGGGGT[C/G]CCTGCCATGTCTCCCCGCTCATCCC
rs73226608	A/G	CCATTGCACTCCAACCTGGACAACA[A/G]AGCAAGACCTTGTCTCAAAAACAAA
rs73226617	A/G	GTAAATATTAGAGTTCTCAAGAAGA[A/G]TCACTTTCATTTATTTATTCTTTCA
rs73226619	G/A	TTTTCTCAGGCCTGGAGAACAACCA[A/G]AGGCTTCAAGGCCTCAGTCTGCGTT

Suplementary Table 9. Statistical associations for previously reported population-wide TB-associated polymorphisms in the Peruvian cohort. Variant are chosen from all variants that reached genome-wide significance in the discovery stage. This include two variants (rs4331426 and rs2057178) reported in west African populations. rs9271378, rs9272785 and rs4733781 reported in the European cohorts.

Chr	SNP	Position(hg19)	Gene	EA	Non-EA	EAF	effect size	standard error	P-value
6	rs9271378	32587300	-	G	A	0.254	2.23E-02	1.28E-02	8.31E-02
6	rs9272785	32610401	HLA-DQA1	A	G	0.665	-3.83E-02	1.35E-02	4.49E-03
8	rs4733781	131296767	ASAP1	C	A	0.33	1.42E-02	1.17E-02	2.27E-01
11	rs2057178	32364187	WT1	A	G	0.036	-2.12E-02	3.03E-02	4.84E-01
18	rs4331426	20190795	CTAGE1	G	A	0.021	5.30E-03	4.13E-02	8.98E-01

Supplementary Table 11. THP1 clones included in RNA-sequencing

THP1 clone number	Sanger sequence (PCR amplified around rs73226617)	Classification
160	TGCAAGATATTGTGCTCAGTGCTAAGAAAGATACAAATATAGAAGGCAATGCTGCTGTTCCAAGGTACATCCAAGGTATATCCTTGGTACAGTAGAGAAGAGATGCTCTTAATGCAAGGTAGAAAATCTGAAGACCACAAAAGATGTCCATGTATATTATTTTGGTAGTTTGGACAAGGGAAACCACAATTCCCTAGGTGACGGGTCAATATTCACCAGATGAGGAGGTAGAAAGAAAGGNNTNGTGGGCTAANAA	Deletion
66	TTGATTGCAAGATGGATCTGTGGTCTCTAGAGCGACTTGGGAAGGCTCTGCGGTTCAGTAGTTTGCATTCTCTTGTCCTCGATTAAAGAGATGCTCTTAATGCAAGGTAGAAAATCTGAAGACCACAAAAGATGTCCATGTATATTATTTTGGTAGTTTGGACAAGGGAAACCACAATTCCCTAGGTGACGGGTCAATATTCACCAGATGAGGAGGTAGAAAGAAAGGGNGGTGGGCTAAA	Deletion
68	TTGATTGCAAGATGGATCTGTGGTCTCTAGAGCGACTTGGGAAGGCTCTGCGGTTCAGTAGTTTGCATTCTCTTGTCCTCGAGAAGAGATGCTCTTAATGCAAGGTAGAAAATCTGAAGACCACAAAAGATGTCCATGTATATTATTTTGGTAGTTTGGACAAGGGAAACCACAATTCCCTAGGTGACGGGTCAATATTCACCAGATGAGGAGGTAGAAAGAAAGGNGTGGTGGGCTAAA	Deletion
79	CTCTAGAGCGACTTGGGAAGGCTCTGCGGTTCAGTAGTTTGCATTCTCTTGTCCTCGATTACTGCAAGTAAGGCTGGGATTCCGTCCTTGGNACATGATATAAGAGATGCGCTTAATGTCTGGNAACCANTCTGAANACCCCTGTATATGTCCTTGTATATTATTTTGGTGGATTGGACAATGGAAACCGCGATTCCCTANGTGACGGGTCATTATTCACCAGATGAAGAGGGGGNAGAACTAGTGGTGGGCTAAA	Deletion
102	TGGATCTGTGGTCTCTAGAGCGACTTGGGAAGGCTCTGCGGTTCAGTAGTTTGCATTCTCTTGTCCTCGATTACTGCAAGTAAGGCTGGTACAGTAGAGAAGAGATGCTCTTAATGCAAGGTAGAAAATCTGAAGACCACAAAAGATGTCCATGTATATTATTTTGGTAGTTTGGACAAGGGAAACCACAATTCCCTAGGTGACGGGTCAATATTCACCAGATGAGGAGGTAGAAAGAAANNNNTGGTGGGCTAAA	Deletion
80	TTTGTTGAAGGCTAGTTTATGCAAGATATTGTGCTCAGTGCTAAGAAAGATACAAATATAGAAGGCAATGCTGCTGTTCCAAGGTGGTACAGTAGAGAAGAGATGCTCTTAATGCAAGGTAGAAAATCTGAAGACCACAAAAGATGTCCATGTATATTATTTTGGTAGTTTGGACAAGGGAAACCACAATTCCCTAGGTGACGGGTCAATATTCACCAGATGAGGAGGTAGAAAGAAAGGGNGGGTGGGCTAATAA	Deletion
124	TCTTTGNTTTATTTTTTCTTANATTGNACACNAGGTGCTCATAAAGCCAGGTATAAAATGTGATGATCCTGTTCCATGGCCCTGGATATTATTTAGAAAAGATGCACTTNGTGCACCGCAATTCCCTAGAAGACCGCTCAATATGCCCCTGATTATTATTTTGAAAGAAAGGAGNGGGGANNTAACAATTCCCTAGGTGACGGGTCAATATTCACCAGATGAGGAGGTAGAAAGAAAGGTGTGGTGGGCTANNAAN	Deletion
12	TTATGCAAGATATTGTGCTCATTGCTCTCAAAGATACAAATATNAAAAGTAATGATGGTGTTCCTGGGGTGCCCTTGGGACCTTGGAAAAGAAAAGCTCATATTGCCTTGAAGAAAATCTGAAAACCTCAAAACATGTCCATGTNTATTATTTTGTTATTTTGGACAATGGAAACCACAATTCCCTAAGTGACTGGGCGATATTCCCCTAATCACCAGATNAAAAGAANGNGNGGAAGGGGGNNAANNGGNNTTAN	Edited without deletion
15	GTGCAAGGAAACTGNACATGCAACCCCCCCAACCCTCCCCATCTTTGCTTCCCCCTGCTGACCCCTGGACTAGCCCTGCTGTCCTTGANACGCTCTAAAAGAAATGCTCTTAATGCGAGGAACAAAATCTAAAGACCACAATATATGTCCGTGTATATTATTTTGGTANTTTGGACTACGCAAACCGCNNTTCCCTAANTGACNNGACAANNTNCNCCANATAANGANNNNAGNGATANNGNNGGTGGGGCTAANN	Edited without deletion
20	GGTAGGTTATGCTATACATTGTGNTGTGTGCTATGATAAAAACNAATATAATATGCAATGCTGCGGTTCCGATCCGTGCTTGTTGCAACAGAGAAGAGAAGATCTTCTTGCAGCGTAGAAAATCANATGACCACCACACAAGACCTTGTTTTTATTTTNTTGGTATGNAGNACNNAAGCCACCACTCCCTACGAGANGAGACNNCATTNTNCACATGANGAGGANGAANAAAAAGAGNGGTGGGNNTNNNATANN	Edited without deletion
24	GCTAGTTTATGCAAGATATTGTGCTCAGTGCTAAGAAAGATACAAATATAGAAGGCAATGCTGCTGTTCCAAGGTGTCCTTGGTACAGTAGAGAAGAGATGCTCTTAATGCAAGGTAGAAAATCTGAAGACCACAAAAGATGTCCATGTATATTATTTTGGTAGTTTGGACAAGGGAAACCACAATTCCCTAGGTGACGGGTCAATATTCACCAGATGAGGAGGTAGAAAGANNNGNNNGGGGGGGGNNNTNNAAA	Edited without deletion
125	ATCTTTGATCAAAGATTTGTTATACGCTATATTGTGCTCAATGTTGAGATCAATGCTAATAAAGAAAGCAATGTTGATGGCCTTGCTGCTGTTCAAAAAAAAAGGTCCTTATGGCAAGGAAAAAAACCTAANAACNCCAAANAAGGCCNTGGNTNNTANTTTNGNAATTTNGAACAAGGAAACCCCCATTTCCTNNGNNAACGGGCCATATTNNNCNNAATAAGAAGGAAAAAGAAAAGNGNNGGTGGGNNNNNAAN	Edited without deletion
131	GATAGCTAATTTATGATATAGATTGCGCTGCTTGCAAANANACATACATATATNGAANGGCTNGNNGNNNNNNCNANGGGNCCTNGNNCCNNAAAAAAAAAAAATGNNNTNANNGNANGGAAAAAAAANNNAANAACNNCAAANAANNNCCNNNNNNTTTATTTNGNNAGTTGGAANAAGGAAANCNNCANTTCCTNAGNNNANGGNNCANNNTNCNCCNANNAAGNAGGAAAAANNANNGGGNGGGGNNTTANNAAN	Edited without deletion
133	GGTATGCTTAAGCTTGAGCTCGTGGCCAATGCTAATACAGATACAAAAAGCAAAGGTGCTGCTGCTGGGCGTCCGTGGCCCTGTNNAAAAAAAAAGCACATGCTGTTAGGGCAAAGATCAGAATACCACNACCAATGTCCATGTCCATGATTTTGATTTTGTGAACTTGGAAAACNNCNATTNCCTATNNNANGGNNANNTATTCACCTTATCANGATGAAGAANNANANNGNNNGGGGNNNNNNGGNNNTNNAAN	Edited without deletion
135	TTGGTNNAAGGTNNNTTNNNNNANAANTTGNGNNNNNNGGTTANAAANAANNNAANNTNNAAAGGNANGNNNNNGNTCCNNNNGCNCCNNNNNACANANNNNNNNANNNNNNNNNNNNGNATNNNNANNNNNNNNNANANNNNNANNNANNNNANTGNNNNNNNNNNNNNNNNNNNNNNNNNNNNNNNNNNACNNNNNNNNNNNNNNNNNNNNNNNNNNNNNNNNNNNNNNNNNNNNNNNNNNNNNNNNNNTN	Edited without deletion
114	AGGCTAGTTTATGCAAGATATTGTGCTCAGTGCTAAGAAAGATACAAATATAGAAGGCAATGCTGCTGTTCCAAGGTTGTCCTTGGTACAGTAGAGAAGAGATGCTCTTAATGCAAGGTAGAAAATCTGAAGACCACAAAAGATGTCCATGTATATTATTTTGGTAGTTTGGACAAGGGAAACCACAATTCCCTAGGTGACGGGTCAATATTCACCAGATGAGGAGGTAGAAAGAAAGNNNGGGTGGGGCTANNAN	Edited without deletion
8	AGGCTAGTTTATGCAAGATATTGTGCTCAGTGCTAAGAAAGATACAAATATAGAAGGCAATGCTGCTGTTCCAAGGTGTCCTTGGTACAGTAGAGAAGAGATGCTCTTAATGCAAGGTAGAAAATCTGAAGACCACAAAAGATGTCCATGTATATTATTTTGGTAGTTTGGACAAGGGAAACCACAATTCCCTAGGTGACGGGTCAATATTCACCAGATGAGGAGGTAGAAAGAANNNNNNNNNGNGGGGCTANAAA	Wildtype
13	GCTAGTTTATGCAAGATATTGTGCTCAGTGCTAAGAAAGATACAAATATAGAAGGCAATGCTGCTGTTCCAAGGTGTCCTTGGTACAGTAGAGAAGAGATGCTCTTAATGCAAGGTAGAAAATCTGAAGACCACAAAAGATGTCCATGTATATTATTTTGGTAGTTTGGACAAGGGAAACCACAATTCCCTAGGTGACGGGTCAATATTCACCAGATGAGGAGGTAGAAAGAAAGNNNNNNGNGGGGCTANAAAN	Wildtype
14	AGGCTAGTTTATGCAAGATATTGTGCTCAGTGCTAAGAAAGATACAAATATAGAAGGCAATGCTGCTGTTCCAAGGTGTCCTTGGTACAGTAGAGAAGAGATGCTCTTAATGCAAGGTAGAAAATCTGAAGACCACAAAAGATGTCCATGTATATTATTTTGGTAGTTTGGACAAGGGAAACCACAATTCCCTAGGTGACGGGTCAATATTCACCAGATGAGGAGGTAGAAAGAAANNGNNGGGTGGGGCTANNAA	Wildtype
18	GGCTAGTTTATGCAAGATATTGTGCTCAGTGCTAAGAAAGATACAAATATAGAAGGCAATGCTGCTGTTCCAAGGTGTCCTTGGTACAGTAGAGAAGAGATGCTCTTAATGCAAGGTAGAAAATCTGAAGACCACAAAAGATGTCCATGTATATTATTTTGGTAGTTTGGACAAGGGAAACCACAATTCCCTAGGTGACGGGTCAATATTCACCAGATGAGGAGGTAGAAAGAAAGNNNNNGGTGGGGCTANANAN	Wildtype
116	GGCTAGTTTATGCAAGATATTGTGCTCAGTGCTAAGAAAGATACAAATATAGAAGGCAATGCTGCTGTTCCAAGGTGTCCTTGGTACAGTAGAGAAGAGATGCTCTTAATGCAAGGTAGAAAATCTGAAGACCACAAAAGATGTCCATGTATATTATTTTGGTAGTTTGGACAAGGGAAACCACAATTCCCTAGGTGACGGGTCAATATTCACCAGATGAGGAGGTAGAAAGAAAGGNGTGGGTGGGCTANNNNAN	Wildtype
122	AGGCTAGTTTATGCAAGATATTGTGCTCAGTGCTAAGAAAGATACAAATATAGAAGGCAATGCTGCTGTTCCAAGGTGTCCTTGGTACAGTAGAGAAGAGATGCTCTTAATGCAAGGTAGAAAATCTGAAGACCACAAAAGATGTCCATGTATATTATTTTGGTAGTTTGGACAAGGGAAACCACAATTCCCTAGGTGACGGGTCAATATTCACCAGATGAGGAGGTAGAAAGAAANGGNNNGNNGGGGCTANAAN	Wildtype
123	AGGCTAGTTTATGCAAGATATTGTGCTCAGTGCTAAGAAAGATACAAATATAGAAGGCAATGCTGCTGTTCCAAGGTGTCCTTGGTACAGTAGAGAAGAGATGCTCTTAATGCAAGGTAGAAAATCTGAAGACCACAAAAGATGTCCATGTATATTATTTTGGTAGTTTGGACAAGGGAAACCACAATTCCCTAGGTGACGGGTCAATATTCACCAGATGAGGAGGTAGAAAGAAAGGNNNGGGTGGGGCTAANAN	Wildtype

Supplementary Table 12. Previously reported TB related genes for cusmized array design.

Gene_name	Type	PubMEDID
XPO1 - RPS29P10	GWAS	20694014
PARD3B	GWAS	20694014
RPL6P14 - TET2	GWAS	20694014
STXBP5	GWAS	20694014
AHCYL2	GWAS	20694014
CYCSP22 - PXDNL	GWAS	20694014
RNA5SP272 - RIPK2	GWAS	20694014
ASAP1	GWAS	25774636
RCN1 - WT1	GWAS	22306650
GLRX5 - TCL6	GWAS	20694014
RNA5SP430 - RPL18P13	GWAS	20694014
CDH13, LOC101928446	GWAS	20694014
DUSP14	GWAS	20694014
RPS4XP18 - UBE2CP2	GWAS	20694014
MC4R - MRPS5P4	GWAS	20694014
ZNF229	GWAS	20694014
IFNGR1	suscptibility to mycobacterial diseases	24821915
IFNGR2	suscptibility to mycobacterial diseases	24821915
IL12B	suscptibility to mycobacterial diseases	24821915
IL12B1	suscptibility to mycobacterial diseases	24821915
STAT1	suscptibility to mycobacterial diseases	24821915
IRF8	suscptibility to mycobacterial diseases	24821915
ISG15	suscptibility to mycobacterial diseases	24821915
NEMO	suscptibility to mycobacterial diseases	24821915
CYBB	suscptibility to mycobacterial diseases	24821915
MR	candidate genes	22825450
CD209	candidate genes	22825450
CLEC7A	candidate genes	22825450
TLR1	candidate genes	22825450

TLR2	candidate genes	22825450
TLR4	candidate genes	22825450
TLR8	candidate genes	22825450
TLR9	candidate genes	22825450
TIRAP	candidate genes	22825450
CR1	candidate genes	22825450
NOD2	candidate genes	22825450
CD14	candidate genes	22825450
P2X7	candidate genes	22825450
VDR	candidate genes	22825450
SP-A1	candidate genes	22825450
SP-A2	candidate genes	22825450
MBL	candidate genes	22825450
TNF	candidate genes	22825450
IL1B	candidate genes	22825450
IL6	candidate genes	22825450
IL8	candidate genes	22825450
IL10	candidate genes	22825450
IL18	candidate genes	22825450
CCL1	candidate genes	22825450
CCL5	candidate genes	22825450
CXCL10	candidate genes	22825450
NOS2	candidate genes	22825450
SLC11A1	candidate genes	22825450
HLA-DRB1	candidate genes	16916662
HLA-DQB1	candidate genes	16916662
TOX	linkage study	23415668
PTX3	GWAS supplementary	20694014
HLA-DQA1	GWAS supplementary	20694014
MBL2	GWAS supplementary	20694014
UBE3A	GWAS supplementary	20694014

CCL18	GWAS supplementary	20694014
CCL4	GWAS supplementary	20694014
IL12RB1	GWAS supplementary	20694014
MC3R	GWAS supplementary	20694014
ULK1	candidate genes	27485354
Notch4	candidate genes	29228365
TOLLIP	candidate genes	22778396
AIM2	candidate genes	22695634

Supplementary Table 13. Sample QC summary. Individuals were excluded if they were missing more than 5% of the genotype data, had an excess of heterozygous genotypes (3.5 standard deviations (s.d.)), duplicated with identity-by-state >0.9 or did not fit early TB progressor selection criteria.

Criteria	#_failed_QC_samples
**missngness >5%**	1
**duplicate individuals**	3
**heterzygosity rate (+- 3.5 s.d)**	14
**old at age of diagnose in cases**	5

Supplementary Table 14. GWAS pre-imputation variant QC summary.

Criteria	#_failed_QC_markers
excessive missingness (>95%)	9,779
different genotype call rates between cases and controls (P<1e-5)	25
HWE P<1e-5 in controls	4,009
duplicated position markers	443
batch effect (P<1e-5)	20,965
Total	35,302

Supplementary Table 15. Used high-LD regions in human genomes (in Grch37).

chr	start	end
1	48000000	52000000
2	86000000	100500000
2	134500000	138000000
2	183000000	190000000
3	47500000	50000000
3	83500000	87000000
3	89000000	97500000
5	44500000	50500000
5	98000000	100500000
5	129000000	132000000
5	135500000	138500000
6	25500000	33500000
6	57000000	64000000
6	140000000	142500000
7	55000000	66000000
8	8000000	12000000
8	43000000	50000000
8	112000000	115000000
10	37000000	43000000
11	46000000	57000000
11	87500000	90500000
12	33000000	40000000
12	109500000	112000000
20	32000000	34500000

Supplementary Table 16. Summary statistics of the top associated SNP, rs73226617, identified in the TB progression GWAS using different genetic relationship matrix.

Method	Effect size	Standard error	P-value
GCTA	0.167	0.030	3.86E-08
REAP	0.167	0.030	3.86E-08
PCRELATE	0.168	0.030	2.49E-08
GEMMA	0.165	0.030	3.93E-08

Supplementary Table 17. Bayesian meta-analysis of rs73226617 to test whether the reported association is restricted to the early progressors with varying prior values.
Values are the disease specific approximate Bayes factor (ABF) for the top associated variant (rs73226617).

**sigma/rho**	**0**	**0.1**	**0.2**	**0.3**	**0.4**	**0.5**	**0.6**	**0.7**	**0.8**	**0.9**
**0.1**	0.380	0.339	0.308	0.284	0.265	0.252	0.244	0.244	0.260	0.327
**0.2**	0.525	0.505	0.485	0.465	0.444	**0.421**	0.396	0.369	0.344	0.333
**0.3**	0.727	0.712	0.694	0.670	0.642	0.608	0.567	0.517	0.457	0.388
**0.4**	0.942	0.929	0.909	0.881	0.845	0.800	0.743	0.671	0.581	0.463

Supplementary Table 18. Summary and accession information for Chip-seq of master transcription factors used in the IMPACT analysis (Figure 3e).

Transcription Factor	Total nucleotides covered	Total peaks	Mean nucleotides per peak	NCBI GEO/ ENCODE Accession
T-BET	57,708,437	51069	1821	GSM2176974, GSM2176976, GSM1527682
GATA3	5,861,945	23,742	246	GSM1859075
STAT3	3,402,600	5,681	622	GSM2545819
FOXP3	1,354,127	9,847	157	GSM1056936, GSM1056937
STAT5	293,719	2,281	129	GSM1056923, GSM1056922
IRF5	5,020,855	7,640	657	GSE38567
IRF1	2,332,588	7,013	333	GSM1057026
**CEBPB**	2,177,346	13,694	159	GSM785496
PAX5	5,426,269	15,927	341	GSM1086293, GSM1086294, GSM1086295, GSM1086296
HNF4A	12,395,645	50,421	380	GSM803460
NA PolII in lymphocytes	38,815,231	71,879	540	GSM1527695, GSM1527697, GSM486494
TCF7L2	13,397,935	27694	484	GSM816438
RXRA	8,478,764	13,186	643	GSM1010767
REST	6,309,445	16,404	384	GSM803335

Supplementary Table 19. Double stranded probe sequences used for Electrophoretic Mobility Shift Assay (EMSA).

RSID	EMSA probe sequence (forward)	EMSA probe sequence (reverse)
rs146526750: C	AGGCGCCCCCCACCA**C**GCCAGGCTAATTTTT	AAAAATTAGCCTGGC**G**TGGTGGGGGGCGCCT
rs146526750: T	AGGCGCCCCCCACCA**T**GCCAGGCTAATTTTT	AAAAATTAGCCTGGC**A**TGGTGGGGGGCGCCT
rs58538713: A	GGCCTCCTCAAGTGG**A**TCCCTGACCCCCCAG	CTGGGGGGTCAGGGA**T**CCACTTGAGGAGGCC
rs58538713: G	GGCCTCCTCAAGTGG**G**TCCCTGACCCCCCAG	CTGGGGGGTCAGGGA**C**CCACTTGAGGAGGCC
rs73226608: A	CCAACCTGGACAACA**A**AGCAAGACCTTGTCT	AGACAAGGTCTTGCT**T**TGTTGTCCAGGTTGG
rs73226608: G	CCAACCTGGACAACA**G**AGCAAGACCTTGTCT	AGACAAGGTCTTGCT**C**TGTTGTCCAGGTTGG
rs11710569: G	GCCGATTTGATCAAC**G**GGAAGAAAGGGTATC	GATACCCTTTCTTCC**C**GTTGATCAAATCGGC
rs11710569: T	GCCGATTTGATCAAC**T**GGAAGAAAGGGTATC	GATACCCTTTCTTCC**A**GTTGATCAAATCGGC
rs73226617: A	GAGTTCTCAAGAAGA**A**TCACTTTCATTTATT	AATAAATGAAAGTGA**T**TCTTCTTGAGAACTC
rs73226617: G	GAGTTCTCAAGAAGA**G**TCACTTTCATTTATT	AATAAATGAAAGTGA**C**TCTTCTTGAGAACTC
rs148722713: -	GGAGGAAGGAAGGAG**-**GGAAGGAAGGAAGAA	TTCTTCCTTCCTTCC**-**CTCCTTCCTTCCTCC
rs148722713: +GGAA	GGAGGAAGGAAGGAG**(+GGAA)**GGAAGGAAGGAAGAA	TTCTTCCTTCCTTCC**(+TTCC)**CTCCTTCCTTCCTCC
rs189348793: C	AGGGATGGAGGAAGA**C**CTACCAAGCAAATGG	CCATTTGCTTGGTAG**G**TCTTCCTCCATCCCT
rs189348793: T	AGGGATGGAGGAAGA**T**CTACCAAGCAAATGG	CCATTTGCTTGGTAG**A**TCTTCCTCCATCCCT

Supplementary Table 20. Sequences and target locations of synthetic guide RNA samples

Target region	Target location (hg38)	Synthetic guide RNA sequence	Quality score (Deskgen)
rs73226617 5'	chr3: 141,681,650	GTAGGGCTGGAATTCCTGCA	66
rs73226617 near	chr3: 141,681,727	CCATGCCTTGATAAGGTGCA	59
rs73226617 3'	chr3: 141,681,962	GAAGAGATGCTCTTAATGCA	63
