# Supplementary material for: Early progression to active tuberculosis is a highly heritable trait driven by 3q23 in Peruvians
Source: Nat Commun. 2019 Aug 21;10:3765. doi: 10.1038/s41467-019-11664-1 (PMC6704092; doi:10.1038/s41467-019-11664-1)
Supplement: Supplementary file 5 — Description of Additional Supplementary Files [file 41467_2019_11664_MOESM5_ESM.docx]

**Title:** Supplementary Data 1:
**Description:** Supplementary Table 6. Summary statistics of the 11 variants identified in the TB progression GWAS and their association results in the previously published TB GWAS dataset. Variants in red were also genotyped using Taqman assays. Effect size, standard error and p-value are reported from linear mixed models with the genetic relatedness matrix (GRM) as random effects to correct for cryptic relatedness and population stratification between collected individuals. Sex, age (and Native American proportion inferred from the ADMIXTURE analysis (K=6)) were included as fixed effects.

**Title:** Supplementary Data 2:
**Description:** Supplementary Table 8. Reported association between the 3q23 polymorphisms and TB in the Ghanaian, Russian and Icelandic data sets.

**Title:** Supplementary Data 3:
**Description:** Supplementary Table 10. In silico epigenomics and sequence annotations from public database.
