# Supplementary material for: Early progression to active tuberculosis is a highly heritable trait driven by 3q23 in Peruvians
Source: Nat Commun. 2019 Aug 21;10:3765. doi: 10.1038/s41467-019-11664-1 (PMC6704092; doi:10.1038/s41467-019-11664-1)
Supplement: Supplementary file 6 — Reporting Summary [file 41467_2019_11664_MOESM6_ESM.pdf]

nature research

Corresponding author(s): *Double-blind peer review submissions: write DBPR and your manuscript number here instead of author names.*

Last updated by author(s): *YYYY-MM-DD*

Reporting Summary

Nature Research wishes to improve the reproducibility of the work that we publish. This form provides structure for consistency and transparency in reporting. For further information on Nature Research policies, see Authors & Referees and the Editorial Policy Checklist.

Statistics

For all statistical analyses, confirm that the following items are present in the figure legend, table legend, main text, or Methods section.

n/a	Confirmed	
☐	☒	The exact sample size (*n*) for each experimental group/condition, given as a discrete number and unit of measurement
☐	☒	A statement on whether measurements were taken from distinct samples or whether the same sample was measured repeatedly
☐	☒	The statistical test(s) used AND whether they are one- or two-sided<br>*Only common tests should be described solely by name; describe more complex techniques in the Methods section.*
☐	☒	A description of all covariates tested
☐	☒	A description of any assumptions or corrections, such as tests of normality and adjustment for multiple comparisons
☐	☒	A full description of the statistical parameters including central tendency (e.g. means) or other basic estimates (e.g. regression coefficient) AND variation (e.g. standard deviation) or associated estimates of uncertainty (e.g. confidence intervals)
☐	☒	For null hypothesis testing, the test statistic (e.g. *F*, *t*, *r*) with confidence intervals, effect sizes, degrees of freedom and *P* value noted<br>*Give P values as exact values whenever suitable.*
☐	☒	For Bayesian analysis, information on the choice of priors and Markov chain Monte Carlo settings
☒	☐	For hierarchical and complex designs, identification of the appropriate level for tests and full reporting of outcomes
☐	☒	Estimates of effect sizes (e.g. Cohen's *d*, Pearson's *r*), indicating how they were calculated

Our web collection on statistics for biologists contains articles on many of the points above.

Software and code

Policy information about availability of computer code

Data collection	All data was downloaded from related data source after getting the approval. No any other software was used in Data collection.
Data analysis	All software used in the present study are publicly available from the Internet and the corresponding versions were described in detail on the section of Online Methods.

For manuscripts utilizing custom algorithms or software that are central to the research but not yet described in published literature, software must be made available to editors/reviewers. We strongly encourage code deposition in a community repository (e.g. GitHub). See the Nature Research guidelines for submitting code & software for further information.

Data

Policy information about availability of data

All manuscripts must include a data availability statement. This statement should provide the following information, where applicable:

- Accession codes, unique identifiers, or web links for publicly available datasets
- A list of figures that have associated raw data
- A description of any restrictions on data availability

Summary statistics is available through the NHGRI-EBI GWAS Catalog https://www.ebi.ac.uk/gwas/downloads/summary-statistics. Raw and processed RNA-sequencing data from the edited THP1 clones have been deposited in the GEO accession GSE134419 [https://www.ncbi.nlm.nih.gov/geo/query/acc.cgi?acc=GSE134419]. All scripts and data for generating figures presented in the manuscript are available at [https://github.com/immunogenomics/TB_progression_GWAS]

Field-specific reporting

Please select the one below that is the best fit for your research. If you are not sure, read the appropriate sections before making your selection.

☒ Life sciences ☐ Behavioural & social sciences ☐ Ecological, evolutionary & environmental sciences

For a reference copy of the document with all sections, see nature.com/documents/nr-reporting-summary-flat.pdf

Life sciences study design

All studies must disclose on these points even when the disclosure is negative.

Sample size	4,002 samples were genotyped
Data exclusions	Individuals were excluded if they were missing more than 5% of the genotype data, had an excess of heterozygous genotypes (±3.5 standard deviations), duplicated with identity-by-state >0.9 or index cases with age at diagnosis greater than 40 years old.
Replication	The experiment was performed on three independent batches of THP1 nuclear extracts
Randomization	The sampling was generally random from the described cohort.
Blinding	The samples which fit phenotypic definition were collected randomly in sampling.

Reporting for specific materials, systems and methods

We require information from authors about some types of materials, experimental systems and methods used in many studies. Here, indicate whether each material, system or method listed is relevant to your study. If you are not sure if a list item applies to your research, read the appropriate section before selecting a response.

Materials & experimental systems

n/a	Involved in the study	
☒	☐	Antibodies
☒	☐	Eukaryotic cell lines
☒	☐	Palaeontology
☒	☐	Animals and other organisms
☐	☒	Human research participants
☒	☐	Clinical data

Methods

n/a	Involved in the study	
☒	☐	ChIP-seq
☒	☐	Flow cytometry
☒	☐	MRI-based neuroimaging

Human research participants

Policy information about studies involving human research participants

Population characteristics	Information on age and gender are also collected at enrollment.
Recruitment	We enrolled index cases as adults (aged 15 and older) who presented with clinically suspected pulmonary TB at any of 106 participating health centers in Lima, Peru. Pulmonary TB patients have been diagnosed by the presence of acid fast bacilli in sputum smear or a positive M.tb culture at any time from enrollment to the end of treatment. All cultures of the index cases were genotyped using mycobacterial interspersed repetitive units-variable number of tandem repeats (MIRU-VNTR). Within two weeks of enrolling an index patient, we enrolled his or her household contacts (HHCs). The M.tb status was determined using the Tuberculin Skin Test (TST).
Ethics oversight	The study protocol was approved by the Institutional Review Board of Harvard School of Public Health and by the Research Ethics Committee of the National Institute of Health of Peru.

Note that full information on the approval of the study protocol must also be provided in the manuscript.
